# Aptamer–liposome targeted nanotherapeutics for cancer therapy: Bibliometric analysis, recent developments and future perspectives

**DOI:** 10.1016/j.mtbio.2026.102766

**Published:** 2026-01-05

**Authors:** Zhao Gao, Sang Du, Jiarui Song, Yating Gao, Xin Peng, Xin Lin, Shuang E, Yinan Zhao, Shubiao Zhang

**Affiliations:** a Key Laboratory of Biotechnology and Bioresources Utilization of Ministry of Education, School of Life Science, Dalian Minzu University, Dalian, 116600, Liaoning, China; b School of Life Sciences, Tianjin University, Tianjin, 300072, China; c College of Chemistry and Chemical Engineering, Liaoning Normal University, Dalian, 116029, Liaoning, China

**Keywords:** Liposome, Aptamer, Targeted drug delivery, Cancer therapy

## Abstract

As one of the leading causes of death worldwide, cancer has driven the advancement of targeted therapy toward greater precision and reduced off-target effects. Liposomes, with their biocompatibility, tunable properties, and clinical success, are among the most promising nanocarriers, yet their tumor-targeting specificity remains limited. Aptamer-functionalized liposomes provide a synergistic solution by combining selective aptamer–receptor recognition with efficient drug encapsulation, achieving enhanced tumor targeting and controlled release. Recent advances have expanded this platform toward multi-targeting, stimuli-responsive systems, and theranostic applications, thereby extending the potential of conventional liposomes. This review offers an integrated perspective on the structural design, internalization pathways, and therapeutic applications of aptamer–liposome systems across various cancers. Key barriers, including aptamer instability, scalable conjugation, and limited clinical translation, are critically discussed, alongside emerging strategies to address them. The convergence of aptamer targeting and liposomal delivery represents a transformative step toward next-generation nanotherapeutics, offering a paradigm shift in precision oncology by enabling personalized, selective, and multifunctional cancer therapy.

## Graphical abstract

**Figure:**
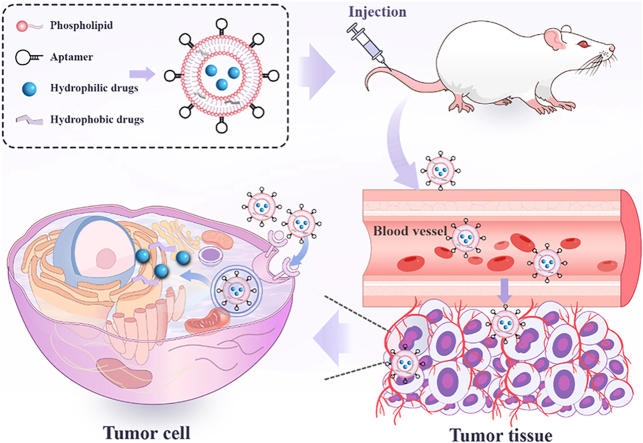

## 1 Introduction

Cancer remains a major challenge in modern medicine, with conventional chemotherapy limited by systemic toxicity and poor precision [[1], [2], [3]]. Advances in nanotechnology and materials science have led to innovative drug delivery systems, among which liposomes represent one of the most clinically validated platforms due to their biocompatibility, versatile drug-loading capacity, and tunable pharmacokinetics [[4], [5], [6]]. Since their discovery in the 1960s by Bangham et al., liposomes, nano-sized vesicles composed of phospholipid bilayers surrounding aqueous cores, have attracted significant attention for drug delivery applications. Their amphiphilic structure enables efficient loading of diverse therapeutics [7,8], while their biodegradability reduces immune clearance [9,10] and systemic toxicity [11]. Surface modifications, most notably PEGylation, further extend circulation time and improve pharmacokinetics, thereby enhancing drug accumulation at tumor sites [[12], [13], [14]]. Clinically, liposomal formulations have achieved remarkable success: for instance, the PEGylated liposomal doxorubicin (DOX) formulation Doxil® significantly reduces cardiotoxicity while maintaining antitumor efficacy [15]. Several other FDA-approved or clinically used formulations, such as Vyxeos [16], Marqibo [17], Onivyde [18], DaunoXome [19], and Lipoplatin [20], further highlight the translational potential of liposomal systems in oncology.

The passive accumulation of liposomes in tumors is primarily attributed to the enhanced permeability and retention (EPR) effect, driven by leaky vasculature and impaired lymphatic drainage [21,22]. However, tumor heterogeneity, poor vascularization, and complex microenvironments often limit EPR-mediated targeting, resulting in inconsistent therapeutic outcomes [23,24]. To address these shortcomings, researchers have developed strategies such as structural optimization [25], surface functionalization [26,27], and stimuli-responsive designs [[28], [29], [30]]. Despite these advances, the inherent constraints of passive targeting underscore the need for active targeting approaches capable of recognizing tumor-specific biomarkers.

Active targeting has been achieved through surface modification of liposomes with ligands such as antibodies [31,32], aptamers [[33], [34], [35]], and small molecules [36,37]. Antibodies provide excellent specificity but are limited by large molecular weight, immunogenicity, high cost, and poor tissue penetration [38]. Small-molecule ligands are easily engineered but often suffer from low affinity and competition with endogenous molecules [37]. Aptamers, in contrast, combine high specificity with favorable physicochemical and pharmacological properties. These short, single-stranded DNA or RNA sequences, typically 20–100 nucleotides in length, fold into unique three-dimensional structures that enable them to bind a broad range of targets, including small molecules, proteins and cells, with antibody-like affinity [39]. Owing to these structural and functional features, aptamers obtained through various selection strategies, most commonly SELEX (systematic evolution of ligands by exponential enrichment)-based approaches, also exhibit practical advantages, such as small molecular size enabling deep tissue penetration, low immunogenicity allowing repeated administration, facile chemical modification, low production costs, and high batch-to-batch reproducibility [40,41]. SELEX-based aptamer selection strategies include protein-based SELEX, cell-SELEX, tissue-SELEX, and in vivo-SELEX [42]. These diversified strategies facilitate the identification of disease-associated biomarkers within more physiologically relevant microenvironments, thereby enhancing aptamer specificity and affinity [43,44]. In addition, they broaden the aptamer repertoire and enable the recognition of cryptic epitopes that are inaccessible to antibodies or small-molecule ligands, further strengthening the potential of aptamers in cancer diagnosis and therapy [45,46].

By combining the molecular recognition precision of aptamers with the protective and versatile drug-loading properties of liposomes, aptamer-functionalized liposomes represent a synergistic platform for targeted tumor therapy. This approach overcomes the limitations of passive targeting, enhances selectivity for diverse tumor biomarkers, and improves therapeutic safety and efficacy. Recent developments have extended these systems into multi-aptamer constructs, theranostic platforms, stimuli-responsive formulations, and codelivery designs, pushing beyond conventional liposomal carriers. Collectively, these innovations hold great promise for precision nanomedicine.

**Fig. 1:**
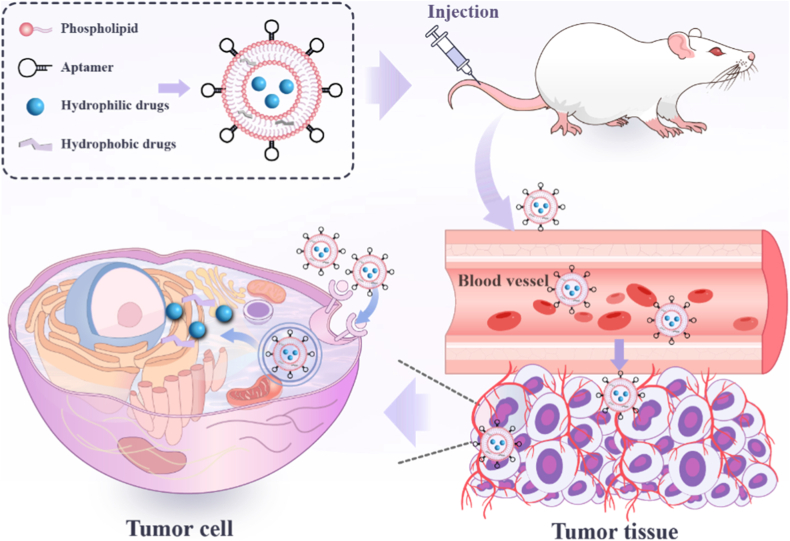
Schematic diagram of aptamer-functionalized liposomes designed for precision-targeted cancer therapy.

Building upon these advances, this review systematically discusses the structural features of aptamers, strategies for their conjugation with liposomes, aptamer-mediated internalization mechanisms, and therapeutic applications across diverse cancer models (Fig. 1). It also critically examines current challenges, such as aptamer stability in vivo, scalable conjugation methods, and clinical translation barriers, and outlines prospective strategies to accelerate the development of next-generation aptamer–liposome nanotherapeutics for precision oncology.

While previous reviews have focused on aptamer selection, conjugation chemistries, and functionalization strategies for aptamer–liposome platforms, and have also expanded to cancer therapy and emerging non-oncology applications such as ocular, skeletal-muscle, and oral delivery systems [47,48], the present review adopts a different perspective. Here, we classify aptamer–liposome nanotherapeutics according to major cancer types, enabling a clearer understanding of target selection, delivery obstacles, and therapeutic outcomes within distinct tumor microenvironments. Moreover, by integrating a bibliometric analysis, we reveal publication trends and the evolution of research hotspots over the past decade. Collectively, this approach provides a focused and comprehensive framework that complements existing literature and offers up-to-date insights into the development landscape of aptamer–liposome therapeutics for cancer.

## 2 Research trends and bibliometric analysis

**Fig. 2:**
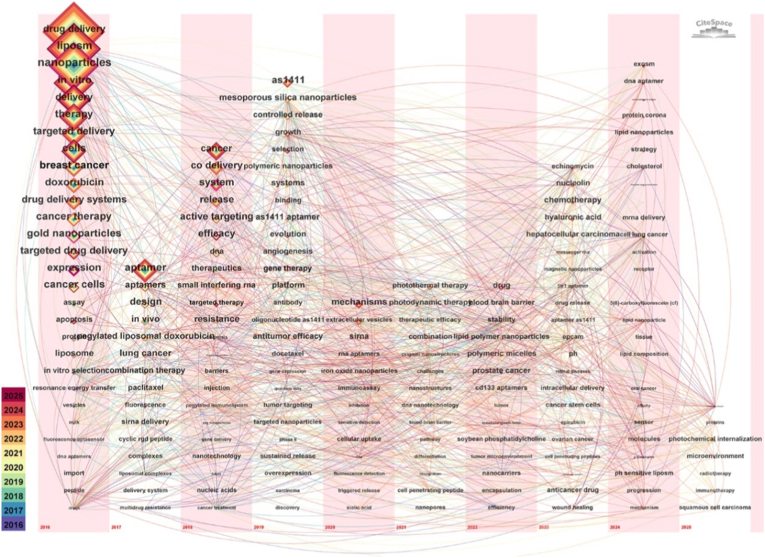
Keyword co-occurrence temporal mapping analysis of published articles.

A bibliometric search was conducted using the Web of Science Core Collection on June 6, 2025, with the query TS = (aptamer∗ AND (liposome∗ OR liposomal)) and the document types restricted to articles and reviews. After removing duplicates and invalid records, 350 valid publications from 2016 to 2025 were included in the analysis. Keyword co-occurrence and clustering were performed using CiteSpace (version 6.3 R3) with standard default parameters. Over recent years, the integration of aptamers with liposomes has attracted growing attention and demonstrated increasing potential in cancer therapy. The 350 retrieved publications reveal a steady rise in yearly output from 2016 to 2025, and the CiteSpace keyword co-occurrence visualization further reflects the evolving research focus within this field (Fig. 2).

In the early phase (2016–2018), research activity was sparse and centered on fundamental concepts such as *aptamer*, *liposome*, and *targeted delivery*, reflecting initial efforts in molecular recognition and carrier design. From 2019 to 2022, the field expanded rapidly, with networks clustering around more functional and application-driven terms such as *controlled release*, *mechanism*, *codelivery*, and *photodynamic therapy*. This shift highlights a transition toward multifunctional systems and translational strategies. In the latest stage (2023–2025), the thematic landscape has become increasingly specialized, emphasizing disease-oriented and microenvironment-focused research, with clusters such as *hepatocellular carcinoma*, *tumor microenvironment*, and *combination therapy*, signaling a clear move toward precision medicine.

The CiteSpace timeline of keyword clusters provides a structured view of this thematic evolution. The most persistent cluster, *cancer therapy*, spans the entire period, consolidating terms related to drug delivery and combination regimens. Early clusters (2016–2018), including *aptamer* and *aptamer-functionalized liposome*, capture the foundational phase of theoretical development. During 2019–2021, clusters such as *nanomedicine delivery* and *aptamer-based therapeutic strategies* expanded significantly, with bridging terms like *AS1411*, *siRNA*, *controlled release*, and *polymeric micelles* marking the transition to functionalization and codelivery paradigms. By 2023–2025, the networks become denser and more disease-specific, highlighting *lung cancer*, *hepatocellular carcinoma*, *microenvironment*, and *efficacy*. Cross-cluster linkages reveal a coherent trajectory: from carrier construction to precise targeting to therapeutic performance. This progression underscores how the field has evolved from foundational research toward application-driven innovation, marking a significant shift toward precision and clinically oriented cancer therapy.

## 3 Structural incorporation of aptamers with liposomes

### 3.1 Aptamer structures and molecular recognition principles

Aptamers are single-stranded DNA or RNA oligonucleotides that can spontaneously fold into defined secondary and tertiary structures in solution, thereby recognizing target molecules with high affinity and specificity [[49], [50], [51]]. Their molecular recognition capability relies on various structural motifs, including stem–loops, bulges, pseudoknots, and G-quadruplexes, which are stabilized through base pairing, hydrogen bonding, π–π stacking, and electrostatic interactions [52]. Stem–loop/hairpin structures represent the most common motifs and are widely involved in nucleic acid–protein interactions [53,54]. Pseudoknots possess more complex topological features and serve as essential structural elements within many ribozymes and viral RNAs [55]. G-quadruplexes are formed by guanine-rich sequences through Hoogsteen hydrogen bonding and exhibit high stability in the presence of monovalent cations. A representative example is AS1411, whose G-quadruplex conformation specifically binds nucleolin, which is overexpressed on the surface of cancer cells [[56], [57], [58]]. Because the biological activity of aptamers is highly dependent on their three-dimensional conformation, any disruption of key structural elements typically leads to a significant decrease in affinity or specificity [51]. Therefore, maintaining structural stability is crucial for their effective application in bioanalysis and targeted drug delivery.

**Fig. 3:**
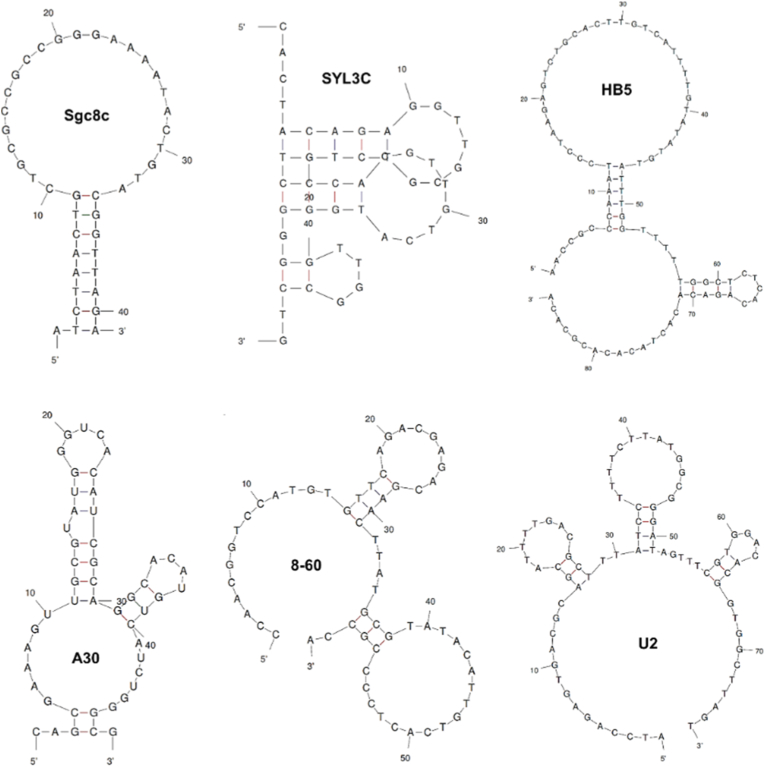
Predicted secondary structures of representative aptamers generated using the Mfold software.

The performance of aptamers in biomedical applications is primarily determined by their nucleotide sequence, tertiary conformations, and their binding affinity to target molecules, typically quantified by the dissociation constant (*K*_d_). Owing to the stringent selection pressures inherent to SELEX, aptamers generated through diverse selection strategies often achieve high binding affinities, underscoring their strong and selective recognition of target molecules. Table 1 summarizes several representative aptamers which are most widely applied in nanotherapeutics for cancer therapy, together with their molecular targets, SELEX methods, and binding affinities. Notably, aptamers such as those targeting nucleolin, PSMA, MUC1, EGFR, PTK7, and PD-L1 exhibit dissociation constants within the nanomolar or sub-nanomolar range, highlighting their advantages for precision targeting. Meanwhile, representative secondary structures of several commonly used aptamers are shown in Fig. 3. These examples demonstrate how distinct three-dimensional conformations and structural motifs endow aptamers with high affinity and specificity, thereby providing a clear mechanistic basis for their targeting capabilities. This structural insight offers valuable guidance for optimizing aptamer modification strategies when engineering aptamer-functionalized liposomal systems.

**Table 1:** Representative aptamers targeting cancer biomarkers.

Aptamer name	Length	Target	SELEX	*K*_d_ (method)	Ref.
S2.2	25 nt	MUC1	protein	0.135 nM (SPR)	[59]
A10–3.2	39 nt	PSMA	cell	2.9 nM (flow cytometry)	[60]
Sgc8c	41 nt	PTK7	cell	0.8 nM (flow cytometry)	[61]
AS1411	26 nt	Nucleolin	cell	34.35 nM (microscale thermophoresis)	[62]
SYL3C	48 nt	EpCAM	cell	38 nM (flow cytometry)	[63]
s5rev	60 nt	CD44	protein	238 nM (microscale thermophoresis)	[64]
CD133-A15	15 nt	CD133	cell	33.9 nM (flow cytometry)	[65]
HB5	86 nt	HER2	protein	316 nM (flow cytometry)	[66]
A30	49 nt	HER3	Protein	45 nM (electrophoretic mobility shift assay)	[67]
8–60	60 nt	PD-L1	protein	1.4 nM (SPR)	[68]
U2	76 nt	EGFR	cell	3.37 nM (ELISA)	[69]

### 3.2 Conjugation strategies for integration of aptamers into liposomes

**Fig. 4:**
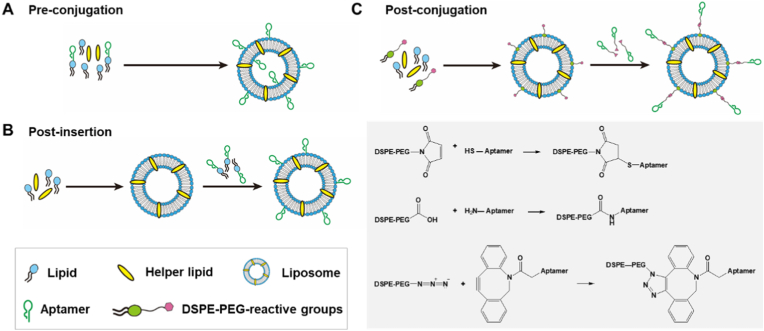
Schematics of strategies for efficiently incorporating aptamers into liposomes, pre-conjugation (A), post-insertion (B) and post-conjugation (C).

The strategies for conjugating aptamers to liposomes can be broadly categorized into two main approaches: one involves introducing the aptamer during the formation of liposomes (pre-conjugation), while the other involves attaching the aptamer after liposome formation through non-covalent or covalent interactions (including post-insertion and post-conjugation) (Fig. 4). The following sections summarize these methods that are widely employed for coupling of aptamers to the surface of liposomes.

#### 3.2.1 Pre-conjugation strategy

The pre-conjugation method, also termed the membrane anchoring strategy, involves covalently conjugating aptamers to lipid components for targeted liposomal modification. In this approach, aptamers are pre-coupled to cholesterol, phospholipids, or other lipid derivatives and incorporated during liposome preparation (Fig. 4A). This enables directional integration of aptamers into the lipid bilayer during vesicle self-assembly [70]. Compared with alternative modification techniques, this method offers distinct advantages include simplified operational protocol, enhanced incorporation efficiency, and structural preservation. However, the main drawback of this method is that the aptamer may be exposed to organic solvents, which could lead to changes in its secondary structure [71,72]. Additionally, some aptamers may become encapsulated within the liposome's inner cavity, occupying space intended for the drug, which can affect the drug loading efficiency. There is also the possibility of interactions between the aptamer and the drug, potentially impacting the drug's effectivenesss [73].

#### 3.2.2 Post-insertion strategy

The post-insertion method involves incorporating aptamers modified with liposomal components (such as cholesterol or phospholipids) into pre-formed liposomes (Fig. 4B). Specifically, when cholesterol-modified aptamers are incubated with pre-formed liposomes at the lipid phase transition temperature, the hydrophobic moiety of cholesterol can spontaneously insert into the hydrophobic core of the liposomal bilayer, while the hydrophilic nucleic acid portion of the aptamer remains exposed on the liposome surface, thereby endowing the liposome with targeted recognition capability. The targeting efficiency is directly influenced by the orientation and surface density of the aptamers on the liposome [41]. It is worth noting that this method may cause slight membrane destabilization, since the process requires heating to the liposome phase transition temperature [74]. The post-insertion strategy exhibits broad compatibility with various aptamers and offers enhanced flexibility for constructing multifunctional and precisely targeted delivery systems [[75], [76], [77]].

#### 3.2.3 Post-conjugation strategy

The post-conjugation strategy involves covalent attachment of aptamers to pre-formed liposomes via reactive lipid anchors embedded in the bilayer (Fig. 4C). Compared with non-covalent interactions, covalent linkage provides superior stability in physiological environments, protecting formulations against pH fluctuations, temperature changes, and biomolecular interference. A critical determinant of success is the choice of linker chemistry, which governs the efficiency, specificity, and durability of aptamer anchoring. Aptamers are typically covalently attached to the lipid bilayer via maleimide**–**thiol coupling, EDC/NHS-mediated amide bond formation, or alkyne**–**azide click chemistry [[78], [79], [80], [81], [82]].

The maleimide**–**thiol reaction is the most widely employed due to its high reactivity under mild conditions, such as room temperature and aqueous buffer system, and its excellent selectivity for thiol groups at physiological pH. This reaction typically yields stable thioether bonds under most experimental conditions [82,83]. In this approach, an aptamer modified with a thiol group is conjugated to DSPE-PEG-maleimide, enabling efficient and site-specific attachment to the lipid bilayer [[84], [85], [86]].

In the EDC/NHS-mediated method, the carboxyl group of DSPE-PEG-COOH is first activated by the addition of EDC and NHS, generating a reactive NHS ester intermediate. Subsequently, an amine-modified aptamer is introduced, which reacts with the NHS ester to form stable amide bonds [87]. This two-step process enables a stable and efficient covalent linkage between the aptamer and the lipid bilayer, thereby enhancing the functionality of the resulting conjugate [78,[88], [89], [90]].

Alternatively, tumor-targeting aptamers could be conjugated to the surfaces of liposomes via click reactions. A classical click chemistry approach involves Cu-catalyzed cycloaddition between an azide and an alkyne to produces a stable 1,2,3-triazole. However, the use of copper is limited by its high cytotoxicity and potential to chelate or interact with biomolecules, complicating quantitative analysis [91]. To overcome these limitations, catalyst-free click reactions have been developed, most notably the strain-promoted alkyne-azide cycloaddition (SPAAC). This approach utilizes strained cyclooctynes (e.g., dibenzocyclooctyne, DBCO) instead of linear alkynes, eliminating the need for copper catalysts [92,93]. For instance, in Kim's study, DBCO-modified DNA aptamers were covalently conjugated to azide groups on PEGylated DSPE-based micelles using copper-free click chemistry [94]. This covalent linkage offers enhanced stability of the formulation in biological environments, minimizing the detrimental effects of pH fluctuations, temperature changes, and the presence of other biomolecules.

### 3.3 Cargo loading strategies for aptamer-functionalized liposomes

Efficient loading of therapeutic cargos is essential for the construction and performance of aptamer-functionalized liposomes. Table 2 provides a comparative overview of different drug types, their corresponding loading strategies, as well as the associated advantages and limitations in aptamer-functionalized liposomal systems. Hydrophilic small-molecule drugs are typically loaded into the aqueous core of liposomes through either passive or active strategies [95,96]. Passive loading methods such as thin-film hydration and reverse-phase evaporation encapsulate the drug during vesicle formation but usually result in low encapsulation efficiency. In contrast, remote loading approaches including pH-gradient, ammonium sulfate gradient and other anion gradient–driven techniques establish transmembrane ionic differentials that promote the diffusion and subsequent trapping of weakly acidic or basic drugs [97,98], thereby achieving significantly higher loading efficiency.

**Table 2:** Comparison of drug types, loading strategies, and their advantages and limitations in aptamer-functionalized liposomal systems.

Cargo Type	Loading Method	Mechanism	Advantages	Limitations in Aptamer-Functionalized Liposomes
Hydrophilic small molecules	Passive loading (hydration, freeze–thaw, reverse-phase evaporation)	Entrapment within aqueous core during vesicle formation	Simple; widely applicable	Membrane perturbation from aptamers may further reduce loading
	Active/Remote loading (pH-gradient, ammonium sulfate, anion-gradient)	Ion/proton gradients drive diffusion and trapping	High EE; stable retention	Aptamer density may affect bilayer packing and reduce gradient stability
Hydrophobic drugs	Lipid-phase co-solubilization	Drug partitions into lipid bilayer during film formation	High loading for lipophilic drugs; stable incorporation	Aptamer insertion may alter bilayer rigidity and influence loading capacity
	Nanoprecipitation-assisted loading	Drug precipitates inside vesicle or within bilayer	Useful for poorly soluble drugs	Sensitive to membrane fluidity perturbation caused by aptamer anchoring
Nucleic acid drugs	Electrostatic complexation with cationic/ionizable lipids	Charge interaction condenses nucleic acids into vesicles	High encapsulation; efficient gene delivery	Aptamer density may cause steric hindrance or alter complexation dynamics
	Polymer-assisted loading (PEI, chitosan, ionizable lipids)	Polyplex formation followed by lipid encapsulation	Protects nucleic acids; enhances stability	Surface aptamers can compete with polymers and affect condensation efficiency
	Layer-by-layer assembly	Nucleic acids intercalated between polyelectrolyte layers	Tunable loading and release	Aptamer modification may interfere with layer formation

Unlike hydrophilic drugs, hydrophobic compounds are incorporated directly into the lipid bilayer during liposome formation. These agents are co-dissolved with phospholipids and, upon hydration, partition into the hydrophobic membrane region, where they remain embedded. Their loading capacity depends on membrane composition and fluidity, which can be subtly altered by surface-conjugated aptamers.

Nucleic acid cargos such as siRNA, miRNA, mRNA and CRISPR/Cas components require cationic or ionizable lipids together with helper lipid to enable efficient condensation and encapsulation [[99], [100], [101]]. Their loading relies on electrostatic interactions between negatively charged nucleic acids and positively charged lipid-based carriers, forming stable complexes that overcome electrostatic repulsion and enhance intracellular delivery efficiency. For nucleic acid cargos, the surface density of aptamers should be carefully optimized because excessive aptamer coverage can introduce steric hindrance, alter surface charge, and interfere with nucleic-acid–lipid complexation [99,102]. Recent studies have shown that tuning the aptamer–lipid molar ratio, applying aptamer post-insertion after nucleic acid loading, adjusting PEG spacer length, and maintaining partial surface coverage (typically ∼2–10 mol%) has been reported in multiple studies to improve nucleic-acid encapsulation and delivery [103,104]. Moreover, controlling surface charge and preventing aptamer clustering have been recognized as important factors contributing to efficient loading and stable liposomal formulations [102].

## 4 Effects of aptamer functionalization on liposomal delivery behavior

Aptamer functionalization has emerged as a powerful strategy to enhance the targeting specificity and therapeutic performance of liposomal drug delivery systems. By decorating the liposome surface with high-affinity, sequence-defined ligands, aptamers enable selective recognition of diseased cells while simultaneously reshaping key physicochemical attributes of the carrier. Such modifications, including changes in particle size, surface charge, and drug encapsulation efficiency, directly influence the pharmacokinetic profile of liposomes, affecting their circulation time, biodistribution, and tumor accumulation [[105], [106], [107], [108], [109]]. Beyond these physicochemical effects, aptamer functionalization also modulates the biological fate of liposomes, guiding their cellular uptake pathways and intracellular trafficking dynamics. Together, these influences highlight the dual role of aptamers in both optimizing formulation properties and orchestrating biological interactions, thereby advancing the precision and efficacy of liposomal therapeutics.

### 4.1 Modulation of physicochemical properties and delivery efficiency by aptamer conjugation

The delivery efficiency of liposomes as drug carriers is influenced by multiple factors, among which aptamer modification plays a critical role. This modification primarily alters the physicochemical properties of liposomes, thereby directly affecting their in vivo delivery performance. These influences can be discussed from three major perspectives.

Firstly, aptamer modification typically increases the particle size of liposomes, and the extent of enlargement often correlates with the aptamer's molecular size [110,111]. Since liposome size critically determines pharmacokinetics, even subtle shifts must be carefully controlled. Liposomes within the 100–200 nm range exhibit more favorable profiles, such as prolonged circulation, sustained drug release, and enhanced tumor accumulation [[112], [113], [114]]. However, once particle size exceeds ∼200 nm, tumor penetration efficiency drops markedly, undermining therapeutic efficacy. Thus, precise control over particle size is necessary to ensure favorable tumor penetration when designing liposomal delivery systems.

Secondly, aptamer conjugation influences the surface charge of liposomes. As negative charged nucleic acid molecules, aptamers typically reduce the zeta potential of liposomes [107,115,116]. The extent of this reduction depends on aptamer surface density. At moderate densities, liposomes retain stability and targeting capability. In contrast, excessively high densities can significantly increase surface negativity, disrupt the protective PEG layer on the liposome surface, and accelerate clearance by the mononuclear phagocyte system, particularly in the liver [117]. Such premature clearance diminishes targeted delivery efficiency, underscoring the necessity of fine-tuning aptamer density. Although no universal standard currently exists, optimal density must be empirically determined for each formulation. For instance, Gu et al. reported an effective density of 10–80 nmol Apt/μmol NPs for PLGA nanoparticles [118], yet corresponding benchmarks for liposomal systems remain to be defined.

Thirdly, aptamer conjugation can influence drug encapsulation efficiency not only by altering bilayer stability but also by modifying the physical organization of the liposomal surface [119,120]. During post-insertion or post-conjugation, aptamers occupy surface area on the outer leaflet and introduce steric hindrance that restricts lipid mobility, leading to subtle disruption of bilayer packing and reduced membrane fluidity. These structural perturbations can weaken transmembrane ion gradients that are essential for remote active loading, and in some cases diminish the internal aqueous volume available for passive loading [119]. Consequently, both passive and active loading efficiencies may decline as the density of surface ligands increases. Recent studies corroborate these effects, demonstrating that nucleic acid or ligand conjugation can reorganize lateral lipid distribution and compromise gradient stability, thereby reducing drug-loading capacity. Therefore, careful selection of conjugation strategies and precise control of aptamer density are crucial to preserve encapsulation performance while achieving effective targeting [121].

By carefully controlling particle size, surface charge, and aptamer density, and by judiciously choosing conjugation strategies, it is possible to maximize delivery efficiency, minimize off-target effects, and achieve superior therapeutic outcomes with aptamer-functionalized liposomal systems. Despite these design considerations, a critical issue lies in quantitatively determining how much of the injected liposomal formulation ultimately accumulates within tumors in vivo.

Although aptamer functionalization enhances the targeting specificity of liposomal nanocarriers, a central challenge in nanomedicine is determining the fraction of the injected formulation that actually reaches tumors. Increasing attention has therefore been directed toward quantitative assessment of in vivo delivery efficiency. To quantify liposome accumulation in tumors, current methodologies are broadly divided into in vivo monitoring and ex vivo quantification. In vivo evaluation typically uses radiotracer-labeled liposomes visualized by PET or SPECT, achieved by incorporating chelator-bearing lipids (e.g., DSPE–DOTA, DSPE–DFO) coordinated with radionuclides such as ^64^Cu, ^89^Zr, or ^99^ᵐTc [[122], [123], [124], [125]]. These formulations enable quantitative whole-body imaging, from which tumor uptake can be derived using standardized uptake values (SUV) or percent injected dose per gram of tissue (%ID/g). Ex vivo quantification enables direct and absolute quantification at the tissue level. γ-counting directly measures radioactivity in harvested tissues, yielding true %ID/g and serving as the reference standard for validating imaging data [126,127]. Alternatively, ICP-MS quantifies metal-labeled nanocarriers such as gold, gadolinium, manganese, platinum in digested tumor samples, enabling highly sensitive determination of the quantity of nanoparticles delivered to the tumor [128].

### 4.2 Role of aptamer functionalization in liposomal cellular internalization

Beyond enhancing targeting specificity, aptamer functionalization profoundly shapes the cellular internalization behavior of liposomal drug delivery systems, making it a key determinant in precision nanomedicine design. The process of uptake generally begins with the high-affinity recognition between surface-displayed aptamers and overexpressed receptors on the target cell membrane [129]. This ligand–receptor interaction triggers endocytic signaling cascades, guiding liposomes into cells via distinct uptake pathways and thereby enabling efficient intracellular drug delivery.

**Fig. 5:**
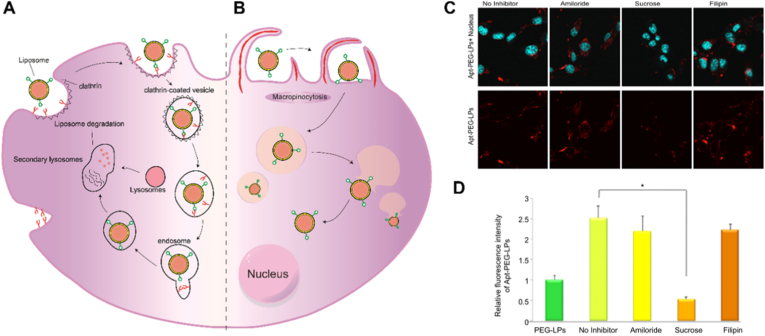
Schematic representation of internalization pathways of aptamers (A) clathrin-mediated endocytosis and (B) macropinosomes. (C) Uptake inhibition assay of Apt-PEG-LPs by different inhibitors. (D) Analysis of cellular uptake of Apt-PEG-LPs based on fluorescence intensity measurements. Adapted from Ara et al., 2014 [131]. Copyright 2014, Elsevier.

Among these pathways, receptor-mediated endocytosis (RME) is considered the predominant mechanism for aptamer-functionalized liposomes. RME relies on the formation of receptor–aptamer complexes, which are subsequently internalized through clathrin-mediated or caveolae-mediated routes, leading to endosome formation and downstream intracellular trafficking (Fig. 5A and B) [34,94,[130], [131], [132]]. For instance, Ara et al. [131] reported that an AraHH001 aptamer-modified PEGylated liposome (Apt-PEG-LP) system achieved efficient tumor targeting primarily through clathrin-mediated endocytosis (Fig. 5C and D).

In addition to RME, macropinocytosis offers an alternative non-specific internalization pathway, particularly relevant in certain cancer cell types. This process involves membrane ruffling and the engulfment of extracellular fluid into large vesicles known as macropinosomes (Fig. 5B), which are subsequently trafficked into the cytoplasm. The AS1411 aptamer, which targets nucleolin aberrantly expressed on the surface of many cancer cells, serves as a representative example. AS1411-induced macropinocytosis has been consistently demonstrated in multiple studies [[133], [134], [135], [136]], and this mechanism has been widely utilized for the intracellular delivery of anticancer therapeutics.

Beyond sequence-specific recognition, the DNA ligands on liposomal carriers can independently influence cellular uptake. Mirkin et al. [102] demonstrated that when DNA strands are densely arranged on liposomal surfaces in a three-dimensional spherical nucleic acid (SNA)-like architecture, even non-targeting, random DNA sequences can significantly enhance the internalization of DNA/liposome conjugates through sequence-independent pathways, which may be associated with recognition by Class A scavenger receptors. These findings indicate that aptamer–liposome systems may exhibit both specific and non-specific uptake pathways, and the contribution of each should be carefully evaluated during mechanistic studies.

In this context, aptamer functionalization not only strengthens liposomal targeting through sequence-specific recognition, but can also modulate delivery efficiency by influencing the dominant pathways of cellular internalization. A comprehensive understanding of both aptamer-mediated and DNA-induced non-specific uptake mechanisms, together with cell-type–specific biological context and therapeutic requirements, provides a robust foundation for the rational design of safe and effective aptamer-functionalized liposomal drug delivery systems.

## 5 Applications in targeted tumor therapy

Malignant tumors remain one of the leading causes of mortality worldwide and continue to present a formidable challenge to medical research [3]. Conventional liposomal drug delivery systems primarily rely on the enhanced permeability and retention (EPR) effect to achieve tumor accumulation. However, the EPR effect is highly heterogeneous among tumor types and across patients, often resulting in inconsistent therapeutic outcomes and unintended off-target toxicity [137]. These limitations highlight the urgent need for more selective and precise therapeutic strategies. In this context, aptamer-functionalized liposomes have emerged as a promising platform for targeted tumor therapy. By integrating aptamers onto the liposomal surface, these nanocarriers gain the ability to recognize tumor-associated markers with high specificity and affinity, while retaining the intrinsic advantages of liposomes such as high drug-loading capacity and favorable biocompatibility. This dual functionality enables precise tumor targeting and efficient drug delivery, features that have been actively explored in preclinical tumor models.

An ideal aptamer-functionalized liposome should meet three essential criteria: high tumor specificity, low immunogenicity, and efficient drug utilization. Together, these characteristics enhance drug accumulation at tumor sites, minimize systemic toxicity, and improve therapeutic efficacy even at reduced drug doses [138]. Mechanistically, aptamers bind to specific receptors on the tumor cell surface and trigger receptor-mediated endocytosis, facilitating liposome internalization. Once internalized, the liposomes are trafficked into lysosomes, where they degrade and release their therapeutic payload, thereby exerting cytotoxic effects on cancer cells [139]. This integrated approach capitalizes on both the targeting precision of aptamers and the delivery efficiency of lipid-based carriers, paving the way for next-generation nanotherapeutics in oncology.

Over the past decades, intensive research efforts have been devoted to identifying aptamers against a wide range of tumor-specific molecular markers. These advances have substantially propelled the development of targeted delivery strategies [140]. A summary of representative aptamers designed for specific tumor targets is presented in Table 3. In the following sections, we will provide a comprehensive review of their applications across different tumor types and critically assess their therapeutic outcomes, aiming to offer a valuable reference for the continued development of aptamer-based targeted cancer therapies.

**Table 3:** Summary of developed aptamer-functionalized liposomes for targeted cancer therapy.

Cancer type	Aptamer	Target	Integration strategy (Linker^a^)	Cargo	Appliance	Ref.
Breast Cancer	HER2 apt	HER2	post-conjugation (EDC/NHS)	Trastuzumab	Improve antitumor efficacy of trastuzumab through the augmentation of the antibody-dependent cytotoxicity effect	[141]
	HER2 apt	HER2	pre-conjugation	Maytansine	Increase drug loading, sensitive tumor probing, and prolonged circulation time	[109]
	HER2 apt	HER2	post-conjugation (maleimide–thiol)	DOX	Selective enhancement of DOX delivery to HER2-positive cells compared with HER2-negative cells	[142]
	HER3 apt	HER3	post-conjugation (EDC/NHS)	DOX	Provide better cell tolerability and efficacy compared with non-targeted delivery	[143]
	SRZ1	4T1 cell	pre-conjugation	DOX	Enhanced tumor-selective delivery and accumulation of DOX in 4T1 tumor tissues	[105]
	CD44 apt, PD-L1 apt	CD44, PD-L1	pre-conjugation	DOX, IDO1 siRNA	Reduce tumor metastasis through a synergistic combination of cancer cell-targeted ICD induction and reversal of the IDO1-mediated immunosuppressive TME	[144]
	anti-CD44 aptamer	CD44	post-insertion	siRNA	Target CD44-expressing tumors and suppressing tumor growth in vivo	[86]
	AS1411	nucleolin	pre-conjugation	DOX	Increase cellular internalization and enhance tumor tissue penetration	[145]
	AS1411	nucleolin	pre-conjugation	DOX	Prevent drug efflux and enhance therapeutic effect	[146]
	AS1411	nucleolin	post-insertion	DOX, mRNA	Increase apoptosis and reduced cancer cell viability.	[75]
	AS1411	nucleolin	post-insertion	Pheophorbide a	Combine photodynamic therapy and chemotherapy, exhibit superior antitumor activity compared to monotherapy	[76]
	AS1411	nucleolin	post-conjugation (maleimide–thiol)	PTX, siRNA	Induce apoptosis of tumor cells and reduce angiogenesis	[84]
Lung cancer	Anti-EGFR apt	EGFR	pre-conjugation	Erlotinib, PFOB	Reverse hypoxia-induced drug resistance, overcome hypoxia-induced erlotinib resistance	[78]
	anti-EGFR apt	EGFR	post-conjugation (EDC/NHS)	Erlotinib	Show binding specificity toward EGFR-mutated cancer cells, enabling preferential delivery to EGFR-mutated tumors	[147]
	Axl apt	Axl	post-conjugation (maleimide–thiol)	1,25-dihydroxyvitamin D3, CTA091	Promote epithelial phenotype, distribute to tumor-bearing lungs in vivo, and increase EGFR TKI activity in vitro and in vivo	[148]
	anti-PD-L1 apt	PD-L1	post-conjugation (maleimide–thiol)	Suberoylanilide hydroxamic acid	Induce the secretion of chemokines that promote the migration of activated T cells into tumor tissues	[149]
	CD133 apt	CD133	post-conjugation (maleimide–thiol)	DTX	Show a significant antitumor activity in A549 tumour mice, with a very low systemic toxicity	[150]
	CD133 apt	CD133	post-conjugation (maleimide–thiol)	Etoposide	Target lung cancer stem cells, causing the aggregation of drugs at the tumor site	[151]
Prostate cancer	A10	PSMA	post-conjugation (EDC/NHS)	^225^Ac	Demonstrate potential for targeted antivascular radiotherapy	[88]
	Anti-PSMA apt	PSMA	post-insertion	DOX	Enhance in vitro cellular binding and target-selective delivery	[152]
	A10	PSMA	post-conjugation (maleimide–thiol)	DTX, siRNA	Precisely target pca cells and effectively deliver DTX and sirna, leading to significant inhibition of pca cell growth and proliferation	[153]
	A10	PSMA	post-insertion	CRISPR/Cas9 targeting PLK1	Show a significant cell-type binding specificity and a remarkable gene silencing effect in vitro	[154]
Hepatocellular carcinoma	TLS1c	MEAR hepatoma cells	pre-conjugation	Cabazitaxel	Enhance MEAR tumor tissue targeting, decrease cytotoxicity and simultaneously retain potent inhibition against tumor growth.	[155]
	APS613–1	GPC3	post-conjugation (maleimide–thiol)	Norcantharidin, Paeonol	Enhance tissue targeting of hepg2, reduce drug toxicity in normal tissues and organs, enable rapid drug release at the target site and improve therapeutic efficacy.	[156]
	CD133 Apt	CD133	post-insertion	DOX, the LCSCs' inhibitor XAV-939	Enhance drug accumulation and penetration in lcscs while reducing nonspecific distribution to normal liver cells and stem cells in other tissues	[106]
	AS1411	nucleolin	post-conjugation (EDC/NHS)	Apigenin	Show significant improvement in cancer cell apoptosis in animal models due to reduced clearance and higher intratumor drug accumulation along with almost nominal toxic effect in liver	[157]
	GT75	eEF1A	post-insertion	Bortezomib, idarubicin	Improve the therapeutic index of the anticancer drugs bortezomib and idarubicin.	[158]
	hEnd-apt	human endoglin	post-conjugation (maleimide–thiol)	CD3 mAb	Enhance antitumoral immunotherapeutic effects	[159]
Colorectal cancer	5TR1	MUC1	post-conjugation (EDC/NHS)	DOX	Enhance tumor-selective delivery and thereby increase DOX accumulation in tumors	[160]
	Syl3c	EpCAM	post-insertion	DOX	Enhance target-specific cellular association with C26 cells and improve the tumor accumulation of DOX	[107]
	EpCAM apt	EpCAM	post-conjugation (EDC/NHS)	miR-139-5p	Inhibit the proliferation, migration, and invasion of one or more CRC cell lines	[161]
	AS1411	Nucleolin	post-conjugation (maleimide–thiol)	Gefitinib	Inhibit tumor cell proliferation	[162]
	AS1411	Nucleolin	post-conjugation (maleimide–thiol)	COL1A1 siRNA	Increase the sensitivity to chemotherapy drugs	[116]
	AS1411	Nucleolin	post-insertion	5-FU	Improve the efficiency, safety, and selective delivery of the drug to colon	[120]
Glioblastoma	A15	CD133	post-conjugation (EDC/NHS)	PTX, siRNA	Induce selectively apoptosis of CD133^+^ glioma stem cells, improve CD133^+^ glioma stem cells' differentiation into non-stem-cell lineages, and inhibit tumorigenesis	[89]
	AS1411	nucleolin	pre-conjugation	Temozolomide, IR780	Target orthotopic gliomas, alleviate tumor hypoxia and consequently reverse resistance of glioma cells to TMZ	[163]
	GBI-10	tenascin-C	post-conjugation (EDC/NHS)	Gadolinium	Increase accumulation of gadolinium at the periphery of C6 glioma cells	[108]
Melanoma	aptATP, aptPD-L1	ATP, PD-L1	pre-conjugation	Auranofin	Enhance the radio-immunotherapeutic efficacy against invasive melanomas.	[115]
	AS1411	nucleolin	post-conjugation (maleimide–thiol)	siRNA	Display excellent tumor targeting capability and significant silencing activity	[164]
	AS1411	nucleolin	post-conjugation (maleimide–thiol)	DOX, ICG	Show higher cytotoxicity and cellular internalization of into B16F0 compared to those samples without AS1411 DNA aptamer	[165]
	endoglin aptamer (ENG-Apt)	endoglin	post-conjugation (maleimide–thiol)	interferon-inducible protein-10 plasmid	Target mouse tumor vascular endothelial cells (mtecs) and enhance cytotoxic T lymphocytes targeting and recruitment to the tumor vasculature	[85]
	Sgc8-c	PTK7	post-insertion	CRISPR/Cas9 plasmid	Improve cellular toxicity and transfection efficiency, higher anti-proliferation activity, remarkable tumor growth suppression	[77]
Cervical cancer	AS1411	nucleolin	post-conjugation (EDC/NHS)	Quercetin	Increase the active targeting and internalization effects	[90]
Acute lymphoblastic leukemia	Sgc8	PTK7	post-conjugation (maleimide–thiol)	Vincristine	Improve the anti-tumor efficacy of VCR and reduce side effects induced by non-specific drug release	[166]
Basal cell carcinoma	AS1411	nucleolin	post-conjugation (maleimide–thiol)	5-FU	Increase the stability of the liposomes and act as a supplementary steric barrier leading to a lower cumulative amount of the released 5-FU	[167]
Choriocarcinoma	Anti-EGFR apt	EGFR	post-conjugation (maleimide–thiol)	SATB1 siRNA	Inhibit the expression of SATB1 in vivo, achieve a high tumor weight inhibitory rate	[168]
Human papillomavirus	AT11	nucleolin	post-insertion	*Origanum vulgare* essential oils	Reduction of ear epithelial cells' proliferation	[169]
Ovarian cancer	AS1411	nucleolin	post-conjugation (maleimide–thiol)	miR-29b	Improve the therapeutic effects of ovarian cancer	[170]

a Linker is applicable only to post-conjugation strategies.

### 5.1 Breast cancer

Breast cancer is the most prevalent invasive malignancy among women and the second leading cause of cancer-related mortality. Its high fatality rate largely stems from delayed diagnosis and the limited efficacy of conventional treatments, such as surgery and chemotherapy, which often cause severe side effects due to nonspecific damage to healthy tissues [171,172]. Risk factors include reproductive history, physiological conditions, dietary habits, and environmental influences [173]. These challenges highlight the need for more precise and targeted therapeutic strategies.

**Fig. 6:**
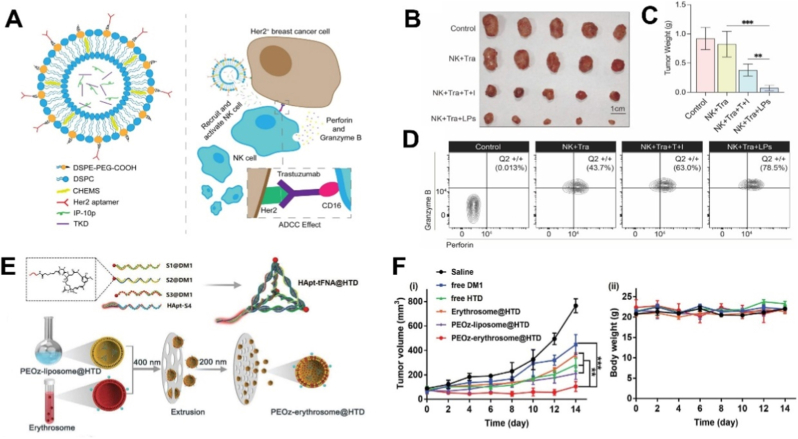
(A) NK cell immunopotentiators-loaded nanoliposomes enhance ADCC effect for targeted therapy against HER2-positive breast cancer. (B) Photographs of excised tumors. (C) corresponding tumor weights from each treatment group (n = 5). (D) Proportion of Perforin-positive or granzyme B-positive rates in tumor-infiltrating NK cells. Adapted from Du et al., 2024 [141]. Copyright 2024, Springer Nature. (E) Schematic illustration of the synthesis of HApt-tFNA@DM1 (HTD), PEOz-erythrosome@HTD, and their proposed antitumor mechanism. (F) Tumor volume and body weight changes in SKBR3 tumor-bearing mice treated with different formulations (n = 5). Adapted from Ma et al., 2022 [109]. Copyright 2022, Wiley-VCH GmbH.

Over the past decades, several receptors, including HER2, CD44, and nucleolin, have been identified as overexpressed biomarkers in breast cancer, making them valuable therapeutic and diagnostic targets [174]. Among these, HER2 is the most extensively studied. Overexpressed in 15–30 % of invasive breast cancers, HER2 plays a central role in cell growth and survival [175]. A representative example is the HER2-I-pHLip system, which combines HER2-specific aptamers with pH-sensitive liposomes to deliver immune-modulating peptides (Fig. 6A–D). This design promoted NK cell recruitment, restored antitumor immune responses, and achieved nearly 90 % tumor inhibition while overcoming trastuzumab resistance, demonstrating strong immunomodulatory potential [141]. Another strategy integrated HER2 aptamers with DNA tetrahedra and a biomimetic membrane, resulting in selective targeting, prolonged circulation, and an inhibition rate of 87.5 % in preclinical models (Fig. 6E and F), with improved biosafety compared to conventional drugs [109]. Additional studies further validated HER2 aptamer–liposome systems as effective carriers, selectively killing HER2-positive cells while sparing HER2-negative counterparts [66,142]. Despite this progress, most evaluations have been conducted in subcutaneous xenograft models, which offer practical advantages but only partially capture the intratumoral heterogeneity, stromal context, and immune complexity of human breast cancer. In addition, evidence regarding long-term biosafety and potential off-tumor effects remains limited, highlighting the need for more comprehensive preclinical assessment across physiologically relevant systems.

CD44, a receptor implicated in adhesion, migration, and metastasis, also represents an attractive target. Apt1-modified liposomes successfully delivered DOX to CD44-positive breast cancer cells while maintaining low systemic toxicity [86]. Building on this platform, subsequent work enabled siRNA delivery with marked gene silencing both in vitro and in vivo [176]. More advanced systems have combined CD44 aptamers with PD-L1 aptamers to co-deliver DOX and IDO1 siRNA. This dual-targeting approach triggered immunogenic cell death and reversed immunosuppression, ultimately producing synergistic chemoimmunotherapy effects [144]. Nonetheless, CD44 is also expressed across various normal cell populations, raising concerns about target selectivity and the risk of unintended biological interactions. Furthermore, most existing studies have not comprehensively examined how the dense and compositionally complex extracellular matrix, together with the dynamic tumor microenvironment, modulates CD44-mediated nanoparticle transport and therapeutic efficacy.

Nucleolin, another multifunctional protein frequently localized on breast cancer cell membranes, has been targeted using the well-known AS1411 aptamer [177]. Early studies showed that AS1411-modified liposomes loaded with DOX improved tumor targeting and cytotoxicity in xenograft models [145]. Recent progress has integrated aptamers with DNA nanostructures; for example, aptamer-functionalized tetrahedral DNA–liposome hybrids achieved significantly enhanced gene delivery efficiency to nucleolin-positive cells, opening new avenues for breast cancer gene therapy [75]. However, the relatively moderate binding affinity and limited in vivo stability of AS1411 may restrict its therapeutic window, and additional optimization is needed to improve nuclease resistance and pharmacokinetic performance [178,179].

Aptamer-functionalized liposomes targeting HER2, CD44, and nucleolin have shown enhanced tumor selectivity, improved pharmacokinetics, and strong antitumor activity with reduced systemic toxicity. Advances such as dual-aptamer designs, DNA nanotechnology, and biomimetic surface engineering further highlight their potential to advance precision nanomedicine in breast cancer. Even so, several biological constraints inherent to breast cancer may limit the consistency of aptamer-mediated targeting: HER2, CD44, and nucleolin exhibit heterogeneous expression across molecular subtypes and can undergo dynamic changes during disease progression or under therapeutic pressure [[180], [181], [182]], while the dense extracellular matrix, stromal remodeling, and immunosuppressive tumor microenvironment characteristic of certain subtypes can impede nanoparticle penetration and attenuate therapeutic efficacy [183,184]. Addressing these challenges will be essential to accelerate the clinical translation of aptamer-guided nanotherapeutics.

### 5.2 Lung cancer

Lung cancer has once again surpassed breast cancer as the most prevalent malignancy worldwide. In 2022, approximately 2.48 million new cases were reported, accounting for 12.4 % of all newly diagnosed cancers, while mortality reached 1.8 million, representing 18.7 % of global cancer deaths [1,185]. Despite progress in screening, biomarker-driven personalized therapies, and early diagnostic technologies, lung cancer remains the leading cause of cancer-related mortality. Traditional chemotherapy, though still widely used, is often accompanied by severe side effects that compromise patients’ quality of life. In this context, targeted therapies, particularly nanocarrier-based approaches, have gained prominence as promising alternatives.

Among molecular targets, the epidermal growth factor receptor (EGFR) plays a central role in tumorigenesis. This transmembrane receptor tyrosine kinase mediates growth factor signaling, and its abnormal overexpression or mutation activates downstream oncogenic cascades, driving uncontrolled proliferation. EGFR alterations are found in approximately 40–80 % of non-small cell lung cancers (NSCLC), especially adenocarcinomas and Asian populations [[186], [187], [188]]. Exploiting this vulnerability, a liposomal nanoplatform (Apt-CL-E) was designed by conjugating anti-EGFR aptamers to chitosan-modified liposomes encapsulating erlotinib [147]. The aptamer enabled selective recognition and binding to EGFR-mutant cells, thereby enhancing tumor-selective intracellular delivery of erlotinib. Compared with non-targeted controls, this system more effectively induced cell-cycle arrest and apoptosis. Expanding on this concept, a multifunctional formulation termed ACLEP was developed, combining erlotinib with perfluorooctyl bromide in aptamer-decorated liposomes [78]. Beyond selective tumor delivery, this dual-functional system alleviated hypoxia, a known driver of resistance, while sustaining drug release, thereby enhancing efficacy. In vivo, ACLEP achieved a tumor inhibition rate of 63.3 %, outperforming conventional liposomes.

**Fig. 7:**
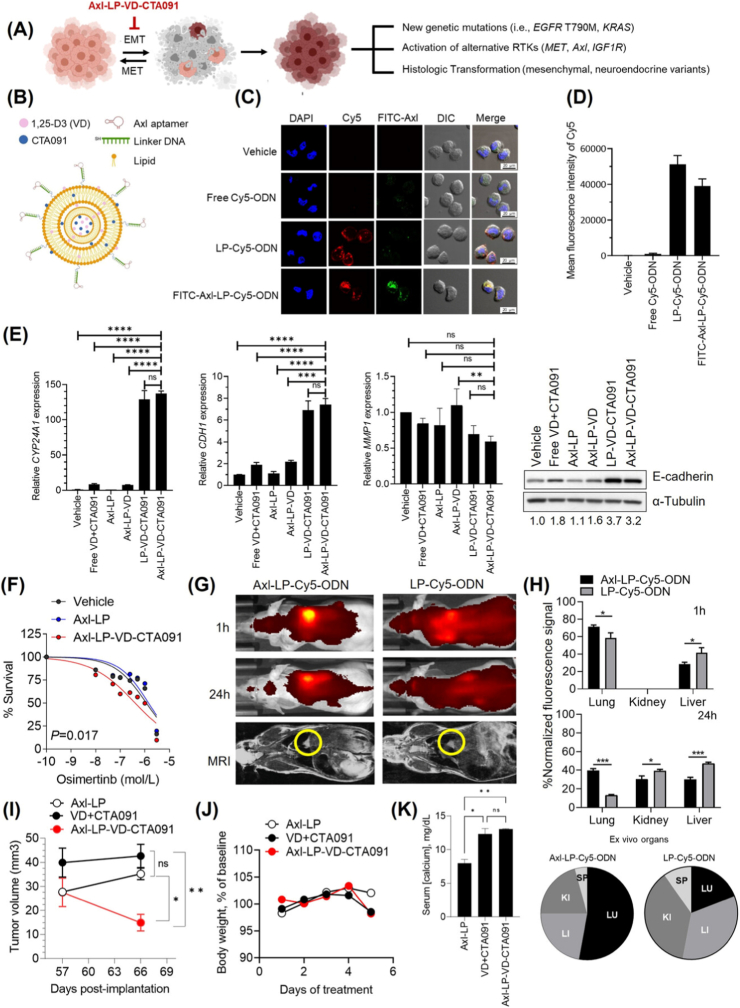
Characterization and biological effects of Axl-LP-VD-CTA091 in EGFR-mutant NSCLC models. (A) Axl-LP-VD-CTA091 targets DTCs undergoing EMT to prevent EGFR TKI resistance. (B) Schematic of Axl-targeted liposomes co-loaded with VD and CTA091. (C, D) Confocal imaging and flow cytometry confirmed selective uptake and binding of Axl-targeted liposomes by H1975OR cells. (E, F) Treatment modulated EMT marker expression and enhanced osimertinib sensitivity. (G, H) In vivo imaging demonstrated tumor-selective biodistribution of Axl-LP formulations. (I–K) Axl-LP-VD-CTA091 suppressed tumor growth in an orthotopic xenograft model without significant toxicity. Adapted from Shaurova et al., 2023 [148]. Copyright 2023, Wiley-VCH GmbH.

Another strategy focused on overcoming resistance to EGFR tyrosine kinase inhibitors (TKIs). To address the persistence of drug-tolerant cells (DTCs), a liposomal system modified with an Axl aptamer (Axl-LP-VD-CTA091) was engineered to co-deliver 1,25-dihydroxyvitamin D3 (VD) and CTA091, a VD degradation inhibitor [148]. In osimertinib-resistant H1975OR cells, Axl-LP-VD-CTA091 showed an 816.5-fold increase in cellular uptake versus background and a 40.9-fold increase versus a free fluorescent probe, indicating efficient internalization. Functionally, it induced an 8-fold upregulation of CDH1 and a 3.2-fold increase in E-cadherin protein, consistent with EMT reversal, leading to a 3.5-fold reduction in osimertinib EC50 (P = 0.004); empty liposomes had no effect. In orthotopic lung tumor models, Axl-targeted liposomes achieved 71 % lung-localized signal at 1 h and 40 % retention at 24 h, compared with 13 % for non-targeted liposomes, corresponding to lung-to-kidney ratios of 2.58 vs 0.5. Enhanced tumor retention translated into potent tumor inhibition, highlighting the therapeutic potential of Axl-guided nanomedicine (Fig. 7). However, Axl expression is heterogeneous across NSCLC subtypes, and its dynamic regulation during therapy-induced tumor evolution may limit the durability of aptamer-mediated targeting. Additionally, the biology of DTCs remains only partially defined, posing challenges for the complete eradication of therapy-resistant cell populations.

Beyond oncogenic signaling, immune evasion remains a major barrier in lung cancer therapy. PD-L1, expressed on 30–60 % of lung tumors, is a key mediator of immune suppression. A PD-L1 aptamer-functionalized liposomal platform was developed to encapsulate the histone deacetylase inhibitor suberoylanilide hydroxamic acid, enabling precise delivery to PD-L1-expressing Lewis lung carcinoma cells [149]. In vitro, the fluorescence signal in LL/2 cells was 1.8-fold higher than that of unmodified liposomes, confirming selective targeting. In vivo, this system achieved a tumor growth inhibition rate of 54.5 %, demonstrating substantial therapeutic benefit through targeted immunomodulation.

Despite these advances, tumor relapse and metastasis remain major clinical hurdles. A growing body of evidence suggests that lung cancer stem cells (CSCs) are central drivers of recurrence and resistance [189]. Markers such as CD133 and EpCAM are commonly used to identify CSCs, providing opportunities for selective targeting [190,191]. CD133-directed liposomal systems have shown particular promise. For example, a DTX-loaded lipid carrier modified with CD133 ligands achieved a tumor inhibition rate of 72 % in murine models [150]. Similarly, an etoposide-loaded CD133-targeted liposome (Lipo@ETP-CD133) displayed strong tumor accumulation and cytotoxicity against lung CSCs [151]. These findings emphasize the importance of integrating CSC-directed strategies into future therapeutic designs.

These findings highlight aptamer-functionalized liposomes offer versatile and precise platforms for lung cancer treatment. By targeting oncogenic drivers such as EGFR, adaptive mechanisms like Axl-driven EMT, immune checkpoints like PD-L1, and CSC-associated markers including CD133, these nanosystems can enhance tumor selectivity, improve drug accumulation, overcome resistance, and synergize with immunotherapy. While challenges remain in aptamer optimization and clinical translation, the preclinical evidence underscores their potential as next-generation nanomedicines for precision lung cancer therapy. Despite these advances, lung cancer presents several unique challenges for targeted delivery compared with other solid tumors. For example, the highly ventilated lung tissue and its dense capillary network can accelerate nanoparticle clearance, limiting sustained accumulation at tumor sites. Moreover, the lung tumor microenvironment is frequently characterized by hypoxia, fibrosis, and immune suppression, each of which can restrict aptamer-guided tumor targeting and overall therapeutic efficacy. Therefore, future studies must systematically address these lung-specific physiological and pathological barriers to accelerate the clinical translation of aptamer-guided nanomedicines.

### 5.3 Prostate cancer

Prostate cancer is the most prevalent malignancy among men over 60 years of age. Although typically slow-growing, about 20 % of cases progress into aggressive, life-threatening disease [192]. Its incidence is shaped by multiple risk factors, including age, race, geography, and family history [193]. Due to this clinical variability, treatment strategies are usually individualized. Androgen deprivation therapy remains the most widely used option [194], yet resistance frequently develops in advanced stages, and conventional chemotherapy is often ineffective. These limitations highlight the need for precise therapeutic approaches that can reduce drug resistance and improve survival [192,193].

**Fig. 8:**
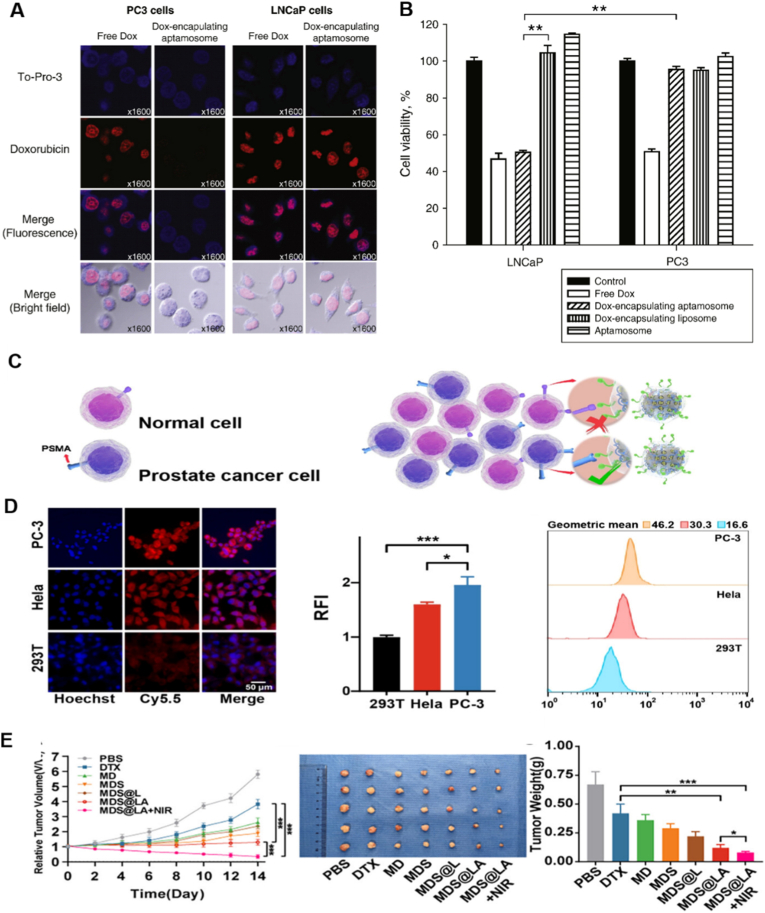
Specific binding (A) and cytotoxicity (B) of Dox-encapsulating aptamosomes to LNCaP prostate cancer cells. Adapted from Baek et al., 2014 [152]. Copyright 2014, Elsevier. (C) Schematic diagram of MDS@LA targeting PC-3 cells. (D) Fluorescence images and quantitative intensity analysis of the selective uptake of MDS@LA by PC-3, HELA, and 293T cells. (E) In vivo antitumor effect of MDS@LA. Adapted from Dai et al., 2025 [153].Copyright 2025, American Chemical Society.

Among available molecular targets, prostate-specific membrane antigen (PSMA) has emerged as one of the most specific and extensively studied biomarkers. PSMA is highly and selectively expressed on prostate cancer cells, with levels far exceeding those in normal or non-prostatic tissues [195]. Exploiting this property, researchers have designed liposomal systems decorated with PSMA-specific aptamers to achieve selective tumor targeting. In one approach, DOX-loaded aptamer-liposomes (aptamosomes) were engineered to bind PSMA-positive cells. Fluorescence studies showed strong intracellular fluorescence in LNCaP cells but not in PSMA-negative PC3 cells (Fig. 8A), confirming PSMA-mediated internalization [152]. Cytotoxicity assays further revealed significantly higher toxicity in PSMA-positive cells compared with non-targeted liposomes, reducing LNCaP cell viability to 50.4 ± 1.09 % versus 104.5 ± 3.99 % for non-targeted DOX-loaded liposomes, while no toxicity was observed from aptamosome lacking drug (Fig. 8B). In LNCaP xenograft models, these targeted carriers accumulated selectively in tumor tissues and resulted in an approximately 92.1 % inhibition of tumor growth relative to untreated controls, with a final tumor volume of 331 ± 195 mm^3^. Compared with non-targeted liposomes, this corresponded to an approximately 54 % reduction in tumor volume (725 ± 235 mm^3^). In addition, the final tumor volume was substantially smaller than that observed for free DOX (1531 ± 280 mm^3^) at an equivalent dose of 0.3 mg/kg.

The PSMA aptamer A10 has also been widely adopted for diverse therapeutic modalities. When coupled to α-particle generators, it enabled targeted radiotherapy [88], while integration into liposomal CRISPR/Cas9 systems allowed precise gene-editing interventions [154]. Building on this foundation, a multifunctional platform based on the enhanced variant A10–3.2 was recently developed to co-deliver DTX and siRNA [153]. In this design, mesoporous polydopamine nanoparticles were loaded with DTX and surface-anchored with siRNA, followed by encapsulation in PEGylated lipid bilayers (Fig. 8C). Conjugation with the A10–3.2 aptamer yielded a targeted nanocarrier (MDS@LA) exhibiting high stability, acid-responsive drug release, and photothermal activity. Markedly higher fluorescence was observed in PSMA-positive PC-3 cells compared with control cells, confirming selective targeting (Fig. 8D). In xenograft models, MDS@LA alone significantly suppressed tumor growth, while combination with near-infrared irradiation further enhanced efficacy, achieving a tumor inhibition rate exceeding 97 % (Fig. 8E).

These advances underscore the therapeutic promise of PSMA-targeted liposomes, which not only improve drug accumulation at tumor sites but also reduce systemic toxicity, thereby enhancing patients’ quality of life. Clinical research in this area continues to advance, yet challenges remain. Current PSMA-targeted systems are less effective against PSMA-negative or drug-resistant tumors. Furthermore, aptamer development against other critical targets, such as the androgen receptor, remains at an early stage [196]. These limitations highlight the need for multi-target strategies that can address tumor heterogeneity and resistance mechanisms. Future innovations in aptamer engineering, particularly those targeting the androgen receptor and additional signaling pathways, will be essential to broaden therapeutic scope and improve precision in prostate cancer management.

### 5.4 Hepatocellular carcinoma

Hepatocellular carcinoma (HCC) ranks as the sixth most common malignancy and the third leading cause of cancer-related mortality worldwide, accounting for over 90 % of all primary liver cancers [3,197]. Owing to its frequent diagnosis at advanced stages, effective clinical management remains a formidable challenge. Although multiple therapeutic agents have been introduced, their efficacy is often limited, failing to produce substantial improvements in long-term prognosis [[198], [199], [200], [201], [202]]. In recent years, nanotechnology has provided innovative strategies for HCC therapy, with aptamer-functionalized liposomes emerging as a particularly promising platform. By enabling tumor-selective recognition through aptamer binding, these nanosystems facilitate precise drug delivery, reduce systemic toxicity, and enhance therapeutic outcomes, offering new hope for patients with advanced disease.

**Fig. 9:**
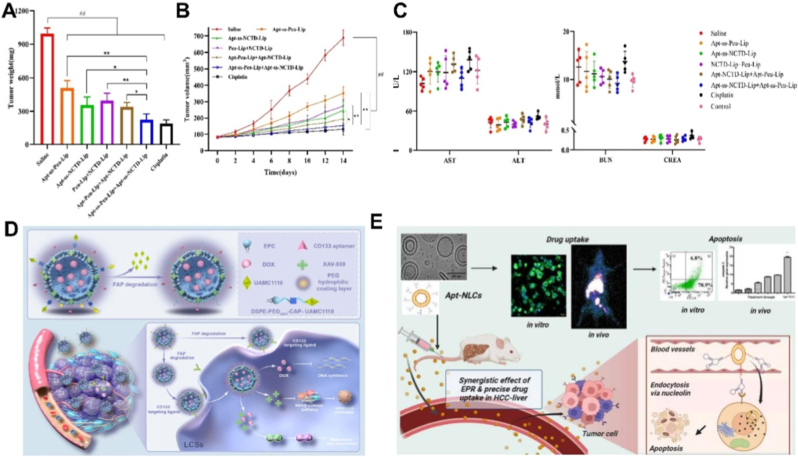
(A) The tumour weights of nude mice at the end of the administration cycle in BALB/C hormonal nude mice. (B) The tumour growth curves. (C) Results of biochemical indexes of the liver and kidney of tumour-bearing BALB/C nude mice in each group. Adapted from Zhang et al., 2025 [156]. Copyright 2025, Elsevier. (D) The schematic diagram shows the anti-HCC mechanism of CAP@CD133-D/X-Lip. Adapted from Kong et al., 2024 [106].Copyright 2024, Elsevier. (E) Schematic illustration of apigenin-encapsulated, PEGylated nanoliposomes functionalized with phosphorothioated amino-modified-AS1411 aptamer. Adapted from Dhara et al., 2023 [157]. Copyright 2023, Springer.

Among the various molecular targets, glypican-3 (GPC3), a membrane-bound heparan sulfate proteoglycan expressed at negligible levels in normal liver but markedly upregulated in HCC, has attracted significant attention [203]. Leveraging this specificity, a redox-responsive dual-drug delivery system was recently designed using the APS613-1 aptamer as the targeting ligand [156]. Redox-sensitive liposomes were loaded with paeonol (Pae) and norcantharidin (NCTD), achieving a tumor inhibition rate of 73.7 %, markedly surpassing single-drug or non-redox formulations. Safety assessments further confirmed no hepatotoxicity or nephrotoxicity, underscoring the clinical potential of this approach (Fig. 9A–C).

Beyond GPC3, other cell-surface receptors such as CD133, nucleolin, and EpCAM also serve as compelling therapeutic targets. As shown in Fig. 9D, A multifunctional nanoliposome (CAP@CD133-D/X-Lip) was engineered to achieve sequential targeting within the HCC microenvironment [106]. By initially homing to cancer-associated fibroblasts and subsequently exposing CD133 aptamers for deeper penetration into liver cancer stem cells (LCSCs), the system enabled codelivery of DOX and the LCSC inhibitor XAV-939. This design disrupted CAF–LCSC interactions, suppressed tumor progression, and achieved an inhibition rate of 83.3 %. Similarly, nucleolin-targeted systems such as PEGylated stealth liposomes functionalized with AS1411 have been employed to deliver apigenin (Fig. 9E), achieving selective accumulation in tumor tissue with minimal hepatotoxicity [157].

In addition to serving as targeting ligands, certain aptamers directly modulate tumorigenic signaling pathways. The GT75 aptamer, for example, binds eukaryotic elongation factor 1 A (eEF1A), an oncogenic protein implicated in HCC progression. Liposomal delivery of GT75 significantly decreased viability of JHH6 cells in a dose- and time-dependent manner and promoted apoptosis, without altering eEF1A expression at the genetic or protein level [158]. Moreover, GT75 enhanced the cytotoxic effects of bortezomib and idarubicin, highlighting its utility both as a direct inhibitor and as an adjuvant to conventional therapies.

Taken together, aptamer-modified liposomes offer a powerful strategy for addressing the major clinical challenges of HCC, including late diagnosis, tumor heterogeneity, and drug resistance. By targeting clinically relevant biomarkers such as GPC3, CD133, and nucleolin, these systems enable efficient drug accumulation in tumor tissues while minimizing off-target effects. Advances in multi-target design, sequential delivery strategies, and aptamers with intrinsic biological activity have already demonstrated strong antitumor efficacy and safety in preclinical models. These innovations establish a solid foundation for clinical translation and position aptamer-functionalized liposomes as a key direction for precision therapy in HCC.

### 5.5 Colorectal cancer

Colorectal cancer, once considered rare, has become one of the most common malignancies, now accounting for about 10 % of all cancer diagnoses and cancer-related deaths worldwide, with nearly 900,000 deaths annually [3,204]. The sharp increase in incidence has been attributed to aging populations, dietary changes, obesity, and smoking [204]. Because early-stage disease is usually asymptomatic, many cases are diagnosed late, leading to poor survival outcomes. Metastasis remains the leading cause of mortality [205]. The integration of targeted therapies into standard regimens for metastatic colorectal cancer has significantly improved patient prognosis [206].

Mucin1 (MUC1), a glycoprotein abundantly expressed in colorectal tumors, has drawn considerable attention as a therapeutic target [207]. One notable strategy employed the MUC1-specific 5TR1 aptamer conjugated to DOX-loaded liposomes (PLD), which showed superior antitumor efficacy in murine colorectal cancer models [160]. In vitro, 5TR1-Doxil showed markedly enhanced cytotoxicity in MUC1-positive C26 cells, with an IC_50_ of 21.05 ± 7.12 μM versus 62.21 ± 6.68 μM for non-targeted liposomal DOX, while no enhancement was observed in MUC1-negative CHO-K1 cells (IC_50_: 56.21 ± 5.66 μM vs. 73.11 ± 4.48 μM). Competitive binding assays confirmed high MUC1 selectivity, as excess anti-MUC1 antibody significantly reduced cellular uptake. In vivo, 5TR1-Doxil significantly delayed tumor growth, extending median survival from 33 days to 50 days and increasing tumor growth delay to approximately 102.8 % compared with conventional liposomal DOX, supporting its potential as an active targeting strategy.

**Fig. 10:**
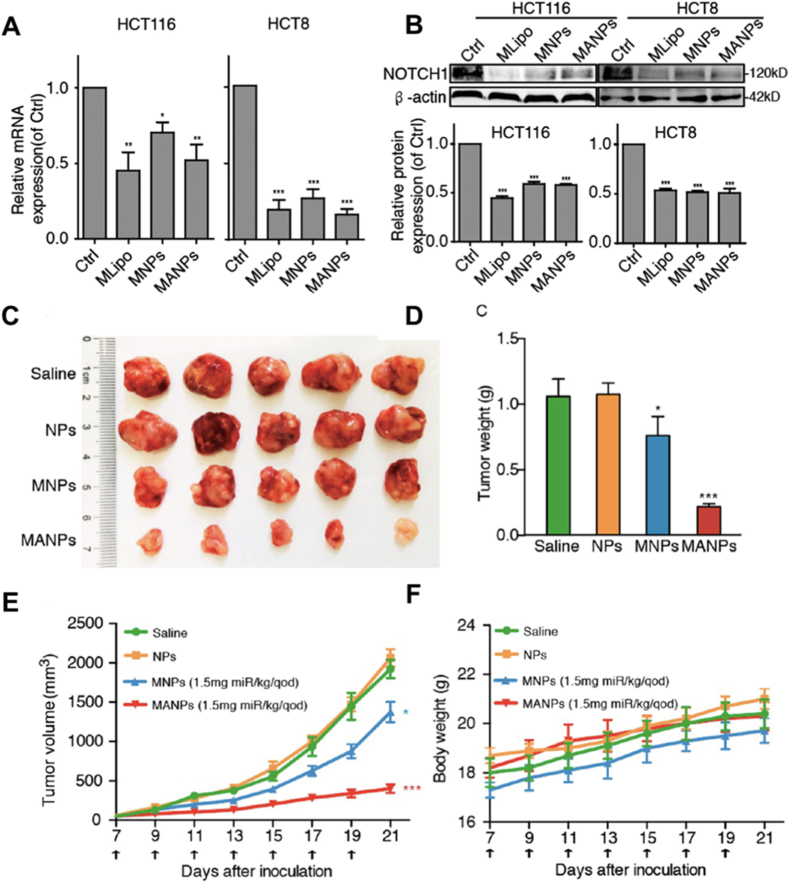
MNPs/MANPs treatment downregulated NOTCH1 expression at both the mRNA (A) and protein (B) levels. (C) Representative tumor images from each group, along with tumor growth curves (D), tumor weight comparisons (E), and body weight monitoring during treatment (F). Adapted from Zhao et al., 2019 [161]. Copyright 2019, American Chemical Society.

EpCAM, another key surface glycoprotein overexpressed in colorectal cancer but minimally expressed in normal epithelia, has also been widely investigated [190]. A DOX-loaded liposomal system modified with the EpCAM RNA aptamer SYL3C (ED-lip) exhibited remarkable therapeutic outcomes in a C26 mouse model [107]. Compared with Caelyx®, ED-lip significantly suppressed tumor progression, prolonged survival, and even achieved complete tumor elimination in several mice. Extending this approach, Zhao and colleagues developed EpCAM aptamer-functionalized cationic liposomes (MANPs) for targeted delivery of miR-139-5p, achieving efficient gene silencing and strong tumor inhibition [161]. Physicochemical characterization confirmed aptamer conjugation with a modest increase in particle size and altered zeta potential, while hemolysis assays demonstrated excellent biocompatibility. Mechanistic studies showed that MANPs effectively downregulated NOTCH1 and EMT-related markers while enhancing E-cadherin expression, thereby inhibiting epithelial–mesenchymal transition (Fig. 10A and B). In xenograft models, MANPs significantly reduced tumor size without systemic toxicity, further underscoring their therapeutic promise (Fig. 10C–F). These results highlight that aptamers play a crucial role in enhancing targeting specificity, making MANPs a potent and biocompatible platform for miRNA delivery. Functionalization with the EpCAM aptamer enables selective tumor recognition and enrichment, thereby improving therapeutic efficacy. Both in vitro and in vivo studies confirmed strong tumor suppression, mainly through precise targeting and EMT regulation [208].

Beyond MUC1 and EpCAM, the nucleolin-binding aptamer AS1411 has also been extensively applied in colorectal cancer nanomedicine. As a structurally stable and easily modifiable DNA aptamer, AS1411 is frequently employed to functionalize PEGylated liposomes, thereby enhancing selectivity and drug accumulation. For example, AS1411-conjugated nanoliposomes loaded with gefitinib achieved superior intratumoral distribution and tumor inhibition in CT26 models [162]. Similarly, AS1411-functionalized liposomes designed for COL1A1 siRNA delivery not only silenced gene expression but also sensitized colorectal cancer cells to DOX, offering a synergistic therapeutic benefit [116]. Further innovation combined AS1411 modification with alginate/chitosan encapsulation to create an oral formulation of 5-fluorouracil (5-FU) with colon-specific release. This design exploited the mucoadhesive and pH-responsive properties of the polymer coating while maintaining aptamer-guided targeting, presenting a novel non-invasive chemotherapy strategy [120].

Advances in aptamer-functionalized liposomal systems for colorectal cancer have shown substantial preclinical promise. By selectively targeting tumor-associated biomarkers such as MUC1, EpCAM, and nucleolin, these platforms enhance drug delivery efficiency, regulate oncogenic pathways, and minimize systemic toxicity. Importantly, several strategies integrate chemotherapy, RNA interference, and oral drug delivery, broadening therapeutic modalities and patient accessibility. Ongoing clinical investigations further underscore the potential of these technologies, positioning aptamer-liposome systems as a vital component of precision colorectal cancer therapy.

### 5.6 Glioblastoma

Glioblastoma (GB) is the most common and malignant primary brain tumor in adults, typically originating from genetic alterations in neural glial cells or their progenitors. Its incidence rises with age, and despite decades of research, effective treatment remains limited [209]. Chemotherapy continues to be the mainstay of GB management [210], but significant side effects and the presence of physiological barriers such as the blood–brain barrier (BBB) and blood–brain tumor barrier (BBTB) greatly restrict its therapeutic efficacy [[210], [211], [212]]. To overcome these obstacles, targeted delivery strategies have been explored, with aptamers emerging as particularly promising owing to their high specificity and ease of modification. These molecules enable precise recognition of glioma stem cells (GSCs) and other tumor-associated components, offering opportunities for improved therapy [213,214].

**Fig. 11:**
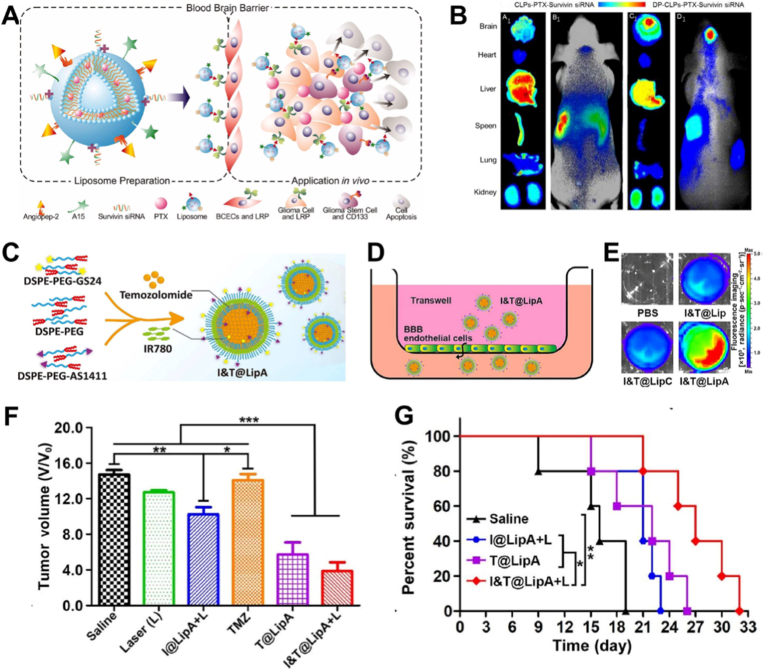
(A) Schematic illustration of DP-CLPs–PTX–siRNA nanocomplex. (B) In vivo fluorescence imaging of intracranial U251-CD133þ glioma tumor-bearing nude mice treated for 24 h with CLPs–PTX–survivin siRNA (B1) or DPCLPs–PTX–survivin siRNA (D1) liposomes, as well as corresponding dissected organs (A1 and C1). Adapted from Sun et al., 2018 [89].Copyright 2018, Taylor & Francis. (C) Schematic illustration of I&T@LipA synthesis. (D) Schematic illustration of the in vitro BBB model. (E) Fluorescence imaging of the bottom chamber of the transwell plate added with PBS, I&T@Lip, I&T@LipC, and I&T@LipA. (F) Quantitative data of body weights of mice and (G) Percent survival of mice in different groups of saline, I@LipA + L, T@LipA, and I&T@LipA + L. Adapted from Zeng et al., 2023 [163]. Copyright 2023, Springer Nature.

One early advance involved a dual-targeting cationic liposomal platform (DP-CLPs) that combined Angiopep-2, a ligand for the low-density lipoprotein receptor-related protein, with the CD133-binding RNA aptamer A15 [89]. Designed for codelivery of paclitaxel (PTX) and survivin siRNA, this nanoplatform demonstrated the ability to cross the BBB, selectively target CD133^+^ GSCs, and enable combined imaging and therapy. In an orthotopic glioma model, systemic DP-CLPs delivering 5 μg PTX and 2.5 μM survivin siRNA efficiently accumulated in tumors and exerted strong anti-GSC activity, inducing GSC apoptosis and differentiation. This resulted in marked suppression of intracranial tumor formation (MRI, day 19) and significantly prolonged survival compared with non-targeted carriers or free drugs, demonstrating the therapeutic potential of this BBB-penetrating, GSC-targeted codelivery strategy (Fig. 11A and B).

More recent studies have extended these concepts into multifunctional designs that combine chemotherapy with photothermal therapy. Zhan and colleagues [163] reported a liposomal system (I&T@LipA) co-encapsulating temozolomide (TMZ) and the photothermal agent IR780. By conjugating aptamers GS24 and AS1411 to DSPE-PEG on the liposome surface, the formulation gained the ability to cross the BBB and selectively target glioma cells (Fig. 11C–E). This dual-functional nanoplatform not only enhanced TMZ efficacy and alleviated tumor hypoxia but also helped overcome TMZ resistance through synergistic chemo–photothermal therapy. The system further exhibited excellent photoacoustic and fluorescence imaging properties, high photothermal conversion efficiency, and good photostability. Both in vitro BBB models and in vivo experiments confirmed targeted accumulation, with orthotopic glioma mouse models showing a tumor inhibition rate of approximately 71 % and significant survival benefits (Fig. 11F and G).

In addition to therapeutic applications, aptamers have also been explored for imaging. A representative example is the GBI-10 aptamer, a single-stranded DNA sequence that binds tenascin-C (TN-C), a protein highly expressed on glioma cells. Gu and collaborators [108] integrated this aptamer onto gadolinium-containing liposomes to develop GBI-10 targeted gadolinium liposomes (GTL). This formulation demonstrated strong potential as a magnetic resonance imaging (MRI) contrast agent for glioma diagnosis in vitro.

Aptamer-based liposomal systems for glioblastoma provide distinct advantages. By recognizing tumor-associated markers such as CD133 and TN-C, aptamers enable multifunctional designs that combine therapeutic and diagnostic capabilities. Preclinical studies confirm their ability to cross physiological barriers, selectively target GSCs, and deliver therapeutic agents or imaging probes, paving the way for personalized therapy and real-time tumor monitoring. However, several challenges remain. Targets such as CD133 are not entirely tumor-specific and may also be expressed in normal stem or progenitor cells, raising concerns about off-target effects. Moreover, research on aptamer-mediated targeting in GB is still relatively limited. Future efforts must focus on discovering more tumor-specific biomarkers and improving both targeting precision and BBB permeability of aptamer-based systems, which will be critical for advancing their clinical applicability.

### 5.7 Melanoma

Melanoma, a malignant tumor originating from melanocytes, is among the deadliest forms of skin cancer [215]. Although its incidence is lower than other skin cancers, it accounts for nearly 90 % of skin cancer–related deaths. The disease is marked by early metastasis, poor prognosis, and resistance to radiotherapy, which together make clinical management particularly difficult [216,217]. Surgical excision remains the standard treatment for primary melanoma, while subsequent strategies, such as immunotherapy, targeted therapy, radiotherapy, or gene therapy, are selected based on disease stage [217]. With the advancement of precision medicine, however, targeted drug delivery technologies have become a central research focus.

**Fig. 12:**
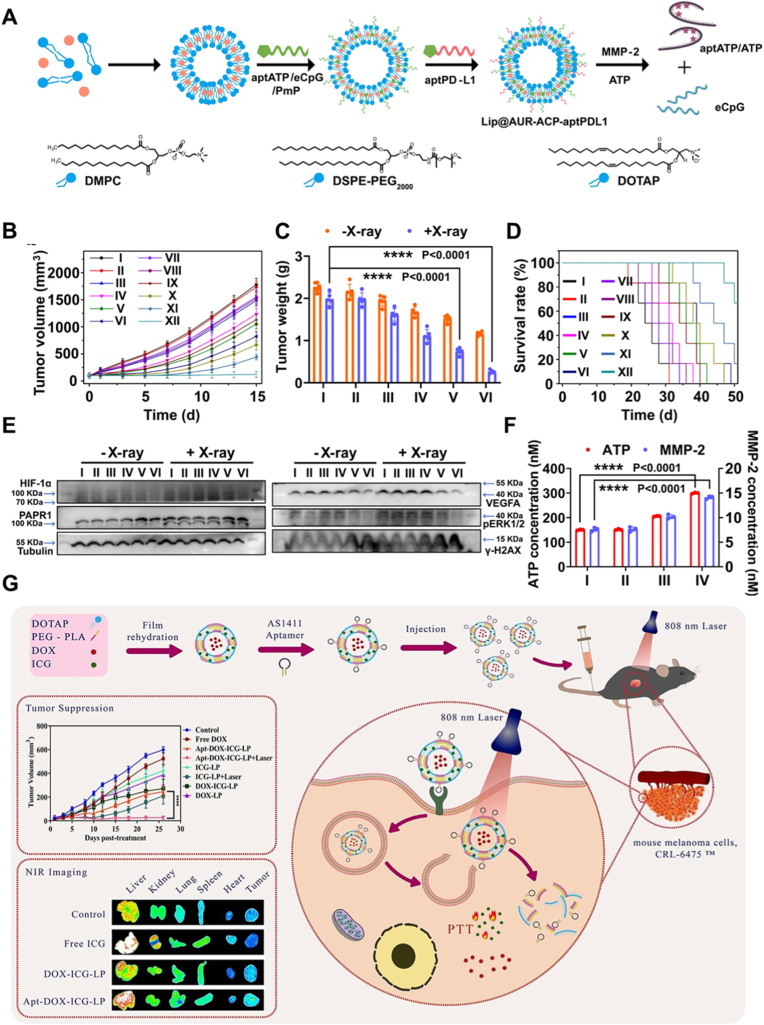
(A) Preparation process and lipid composition of Lip@AUR-ACP-aptPD-L1. (B, C) Tumor volume progression during treatment and final tumor weight analysis across different groups. (D) Survival curves showing that combination therapy significantly prolongs overall survival. (E) Western blot analysis of key protein expression in tumor tissues. (F) Measurement of ATP and MMP-2 release in tumor tissues under various treatments. Adapted from Ren et al., 2024 [115]. Copyright 2024, Springer Nature. (G) Schematic illustration of nucleolin-targeted DOX and ICG co-loaded theranostic lipopolymersome for photothermal-chemotherapy of melanoma in vitro and in vivo. Adapted from Abbasi et al., 2024 [165]. Copyright 2024, Elsevier.

Key aptamer targets in melanoma include PD-L1, nucleolin, and PTK7. By binding to these molecules with high specificity, aptamers show promise in diagnosis, targeted therapy, and combination immunotherapy. One example is a multifunctional fusogenic liposome system (Lip@AUR-ACP-aptPD-L1) designed to overcome PD-L1-mediated immune evasion and enhance radio-immunotherapy in solid tumors [115] (Fig. 12A). This platform integrates three modules: a PD-L1 aptamer for selective tumor recognition; auranofin (AUR) as a radiosensitizer to amplify radiation-induced immunogenic cell death; and a multivariate-gated aptamer assembly (ACP) responsive to ATP and MMP-2 signals in the tumor microenvironment, enabling controlled release of the immunostimulatory agent eCpG. Upon fusion with tumor cell membranes, AUR is delivered intracellularly while the ACP complex remains anchored externally until triggered by ATP and MMP-2, releasing eCpG and activating dendritic cells. Meanwhile, AUR inhibits VEGF signaling, reducing immunosuppressive cell infiltration. In vivo, the combination of Lip@AUR-ACP-aptPD-L1 with low-dose irradiation achieved a tumor inhibition rate of ∼92.3 % and extended median survival beyond 50 days (Fig. 12B–D). Mechanistic analysis confirmed enhanced DNA damage signaling (*γ*-H2AX, PARP1; Fig. 12E) and elevated ATP and MMP-2 levels (Fig. 12F), validating the strong synergistic antitumor effect.

Nucleolin has also been explored as a therapeutic target. A PEGylated cationic liposomal system (ASLP) functionalized with the AS1411 aptamer was used for siRNA (siBraf) delivery, effectively silencing the BRAF gene in melanoma cells with high nucleolin expression and suppressing tumor growth in A375 xenografts [164]. Building on this strategy, a lipid–polymer hybrid nanoparticle (Apt-DOX-ICG-LP) co-loaded with DOX and indocyanine green was developed, also functionalized with AS1411 [165]. Under 808 nm laser irradiation, this system combined targeted chemotherapy with photothermal therapy, producing a tumor inhibition rate of ∼95.3 % in B16F0 melanoma models (Fig. 12G).

PTK7, another melanoma-associated target, was exploited by engineering a hybrid vesicle composed of cationic lipid and mesenchymal stem cell membranes, electrostatically coated with the Sgc8-c aptamer. This Apt-Hybrid system delivered a BIRC5 CRISPR/Cas9 plasmid to achieve gene editing of survivin, a critical anti-apoptotic protein [77]. The approach yielded high transfection efficiency, strong BIRC5 knockout effects, and suppressed tumor proliferation in vitro. In vivo, it achieved ∼75 % tumor inhibition in B16F0 melanoma models, reduced Ki-67 expression, and increased apoptosis, underscoring the potential of aptamers for safe and precise CRISPR-based therapies.

Despite these advances, major challenges remain. Current aptamer targets in melanoma are limited in number, show partial overlap with normal tissues, and are insufficient to address tumor heterogeneity. Furthermore, the absence of standardized clinical systems restricts translation. Future efforts should focus on enhancing aptamer specificity and pharmacokinetics through structural and chemical modifications, while integrating advanced technologies such as membrane proteomics and single-cell sequencing to identify novel melanoma-specific biomarkers. These strategies will be essential for achieving more precise and effective targeted therapies.

### 5.8 Other cancers

Aptamer–liposome delivery systems have been extensively explored in cancer therapy due to their ability to enhance targeting specificity, reduce off-target effects, and improve therapeutic outcomes. While the majority of studies have concentrated on common solid tumors, there is growing interest in their application to rare malignancies and hematologic cancers. Rare solid tumors, although individually infrequent, collectively account for nearly 20 % of all cancer cases worldwide, placing a substantial burden on clinical diagnosis and treatment systems [3,218]. Hematologic malignancies primarily consist of circulating cancer cells and lack a well-defined tumor microenvironment, posing distinct challenges for targeted nanoparticle delivery [219,220]. The following section summarizes recent advances in aptamer–liposome platforms for rare solid tumors and hematologic malignancies, highlighting key findings and remaining challenges.

Progress in developing aptamer-mediated therapies for rare solid tumors has been relatively limited, largely due to the small patient population, challenges in developing representative preclinical models, and constrained experimental and clinical resources. Moreover, research has predominantly focused on broadly expressed pan-cancer biomarkers, such as PTK7, nucleolin, and EGFR [[221], [222], [223]], while studies specifically addressing biomarkers unique to rare cancers remain scarce. Nevertheless, several exploratory studies have begun to demonstrate the therapeutic potential of aptamer–liposome systems in rare solid tumors. In a basal cell carcinoma model, AS1411 aptamer-modified 5-fluorouracil liposomes exhibited markedly enhanced cytotoxicity and higher therapeutic selectivity toward TE 354.T cells compared with unmodified liposomes [167]. Similarly, in choriocarcinoma, a ternary nanocomplex composed of liposomes, polyinosinic–polycytidylic acid, and EGFR aptamer-conjugated DNA (EGFR-LPDS) was designed to deliver SATB1 siRNA. This system efficiently silenced SATB1 expression and induced apoptosis in vitro, while also demonstrating potent antitumor activity in vivo, with a tumor weight inhibition rate reaching 81.4 % in xenograft models [168]. Collectively, these findings indicate that aptamer–liposome platforms hold significant promise for the targeted treatment of rare solid tumors.

Unlike solid tumors, hematologic malignancies involve freely circulating cancer cells and lack a defined microenvironment, complicating targeted nanoparticle delivery. Although liposomes and aptamers have individually demonstrated efficacy in the diagnosis and treatment of hematologic diseases [[224], [225], [226]], their combined application as aptamer–liposome platforms remains largely underexplored. Nevertheless, existing studies indicate promising therapeutic potential for this approach in hematologic malignancies. In an acute lymphoblastic leukemia (ALL) model, sgc8 aptamer-functionalized liposomes were developed to target PTK7, which is abundantly expressed on T-cell precursors. When camptothecin was encapsulated in these carriers, the resulting nanocarriers achieved tumor-specific accumulation in vivo and markedly suppressed tumor growth in mouse models [166]. These results demonstrate that aptamer functionalization can substantially improve drug-targeting specificity and therapeutic efficacy in hematologic cancers. Future studies focusing on selective targeting of cell-surface antigens, optimization of in vivo stability, and enhancement of delivery efficiency may provide new avenues for precision therapy in hematologic cancers.

Overall, accumulating evidence supports the feasibility and potential advantages of aptamer-based delivery systems in both solid and non-solid malignancies. Systematic identification of rare cancer-specific surface biomarkers, in combination with the integration of aptamer-targeting strategies, advanced nanotechnology, and complementary therapeutic modalities such as immunotherapy and photodynamic therapy, is expected to further improve treatment precision, safety, and applicability. Such efforts will likely expand the utility of aptamer–liposome platforms in precision oncology.

## 6 Conclusion and outlook

This review has systematically summarized the structural features, targeting mechanisms, and recent advances in aptamer-functionalized liposomes for cancer therapy. Extensive studies demonstrate that these systems combine the high affinity and specificity of aptamers for tumor-associated biomarkers with the intrinsic advantages of liposomes, such as high drug-loading capacity, protection from degradation, sustained release, biocompatibility, and facile surface modification, thus establishing a powerful platform for active targeted delivery. Unlike conventional passive targeting strategies that rely solely on the enhanced permeability and retention effect, aptamer-liposomes achieve more efficient drug accumulation at tumor sites and substantially reduce off-target toxicity. Preclinical evidence in breast cancer, lung cancer, colorectal cancer, and other malignancies has confirmed that aptamer-liposomes enhance the therapeutic efficacy of chemotherapeutic and nucleic acid drugs, broaden the therapeutic window, and provide a promising strategy to address the limitations of standard chemotherapy, including low selectivity, systemic toxicity, and drug resistance.

### 6.1 Current challenges and barriers

Despite encouraging progress, several obstacles remain before clinical translation can be achieved. First, targeting specificity remains a challenge: many tumor-associated receptors used for aptamer selection are not exclusively expressed in malignant cells, raising the risk of off-target binding. This underscores the need for comprehensive profiling of tumor cell surface receptors and rigorous quality control during aptamer development to ensure reproducibility. Second, in vivo delivery efficiency continues to be a major bottleneck. Even with high-affinity aptamers, liposomal carriers must traverse multiple biological barriers. While microenvironment-responsive designs (e.g., pH-, thermo-, or light-sensitive lipids) can enhance site-specific release, the overall accumulation and release of drugs in deep tumor tissues often remain insufficient. Third, the biocompatibility and biosafety of these systems require more systematic investigation. Key parameters, including aptamer density, particle size, surface charge, and PEGylation, strongly influence circulation time, biodistribution, tumor-targeting efficiency, and clearance. Thus, the establishment of standardized evaluation systems for biological performance and safety is crucial for rational design.

### 6.2 Clinical translation and regulatory considerations

Clinical translation of aptamer–liposome systems remain at an early stage, particularly when compared with approved liposomal formulations such as pegylated liposomal DOX, vincristine liposome (Marqibo®), and the dual-drug liposome CPX-351 (Vyxeos®) used in hematologic malignancies [17,227]. Although aptamers have demonstrated human safety in early-phase trials [228], the combination of aptamer with a complex nanocarrier introduces additional regulatory challenges spanning chemistry–manufacturing–controls (CMC), nonclinical safety, and clinical comparability. Current FDA and EMA guidelines for liposomal products require rigorous control of critical quality attributes and emphasize the need for human pharmacokinetics and bioequivalence studies [229,230]. The recent FDA guidance for oligonucleotide-based therapeutics further highlights sequence-dependent toxicity and immunogenicity risks [231]. Therefore, aptamer–liposome systems require standardized in vivo characterization, including translational PK/PD profiling, immunogenicity and hypersensitivity testing, and risk-based monitoring during repeated dosing, to ensure reproducible safety, efficacy, and regulatory acceptance.

### 6.3 Future perspectives

Interdisciplinary innovation is expected to accelerate progress in this field. Multivalent aptamer engineering may enable simultaneous recognition of multiple receptors, thereby improving specificity. Tumor microenvironment-responsive liposomal designs provide more precise spatial and temporal control of drug release. High-throughput and automated aptamer screening platforms, combined with AI-assisted sequence optimization, offer new opportunities to enhance targeting accuracy while reducing development costs. Furthermore, the integration of aptamers into multifunctional nanoplatforms capable of combining drug delivery with imaging, phototherapy, or immunotherapy, holds great promise for next-generation precision oncology.

In conclusion, while aptamer-functionalized liposomes remain largely exploratory, their exceptional targeting capability, programmability, and compatibility with emerging therapeutic modalities make them an attractive platform for future clinical development. With continued progress in target discovery, delivery optimization, biosafety evaluation, and scalable manufacturing, aptamer-liposome systems are expected to evolve into clinically translatable platforms, paving the way for more precise, effective, and less toxic treatment strategies for malignant tumors.

## CRediT authorship contribution statement

**Zhao Gao:** Writing – original draft, Resources, Investigation, Conceptualization. **Sang Du:** Software, Resources, Methodology. **Jiarui Song:** Visualization, Investigation. **Yating Gao:** Investigation, Conceptualization. **Xin Peng:** Writing – review & editing, Validation. **Xin Lin:** Supervision, Funding acquisition. **Shuang E:** Writing – review & editing, Project administration, Funding acquisition. **Yinan Zhao:** Writing – review & editing, Funding acquisition. **Shubiao Zhang:** Writing – review & editing, Funding acquisition.

## Declaration of competing interest

The authors have declared no conflict of interest.

## Figures and Tables

**Table:** 

EPR	Enhanced permeability and retention
RGD	Arg-Gly-Asp tripeptide
SELEX	Systematic Evolution of Ligands by Exponential Enrichment
EDC	1-Ethyl-3-(3-dimethylaminopropyl) carbodiimide
NHS	N-Hydroxysuccinimide
PEG	Polyethylene glycol
DSPE	1,2-Distearoyl-sn-glycero-3-phosphoethanolamine
SPAAC	Strain-promoted alkyne-azide cycloaddition
DBCO	Dibenzocyclooctyne
PLGA	Poly(lactic-co-glycolic acid)
Lip	Liposome
Apt	Aptamer
RME	Receptor-mediated endocytosis
HER2	Human epidermal growth factor receptor 2
HER3	Human epidermal growth factor receptor 3
ICD	Immunogenic cell death
NK cell	Natural killer cells
DTX	Docetaxel
DOX	Doxorubicin
PD-L1	Programmed Death-Ligand 1
IDO1	Indoleamine 2,3-dioxygenase-1
PPa	Pheophorbide a
PTX	Paclitaxel
EGFR	Epidermal growth factor receptor
TKI	Tyrosine kinase inhibitor
PCa	Prostate cancer
Cab	Cabazitaxel
HepG2	Human Hepatoblastoma G2
NSCLC	Non-small cell lung cancers
DTCs	Drug-tolerant cells
VD	1,25-dihydroxyvitamin D3
EMT	Epithelial to mesenchymal transition
EpCAM	Epithelial Cell Adhesion Molecule
PSMA	Prostate-specific membrane antigen
HCC	Hepatocellular carcinoma
GPC3	Glypican-3
LCSCs	Liver cancer stem cells
MUC1	Mucin1
BBB	Blood-brain barrier
BBTB	Blood-brain tumor barrier
GSCs	Glioma stem cells
TMZ	Temozolomide
TN-C	Tenascin-C
eEF1A	Elongation factor 1 A
mAb	Monoclonal antibody
DOTAP	1,2-Dioleoyl-3-trimethylammonium-propane
Chol	Cholesterol
COL1A1	Collagen 1A1
siRNA	Small interfering RNAs
5-FU	5-Fluorouracil
miR	MicroRNAs
CRC	Colorectal cancer
AUR	Auranofin
ICG	Indocyanine Green
PTK7	Protein tyrosine kinase 7
CRISPR	Clustered Regularly Interspaced Short Palindromic Repeats
TKD	Hsp‐70‐derived 14‐mer peptide
FAP	Fibroblast activating protein
CAFs	Cancer-associated fibroblasts
PK/PD	Pharmacokinetics/Pharmacodynamics
AI	Artificial Intelligence

## Data Availability

No data was used for the research described in the article.
